# Qualitative and quantitative evidence of motivation states for physical activity, exercise and being sedentary from university student focus groups

**DOI:** 10.3389/fspor.2023.1033619

**Published:** 2023-03-21

**Authors:** Matthew A. Stults-Kolehmainen, Todd A. Gilson, Nicholas SantaBarbara, Paul C. McKee, Rajita Sinha, John B. Bartholomew, Daniel Boullosa, Christopher J. Budnick, Fabio Amador Bueno, Adrian Haughton, Jessica L. Barker, Garrett I. Ash

**Affiliations:** ^1^ Digestive Health Multispecialty Clinic, Yale New Haven Hospital, New Haven, CT, United States; ^2^ Department of Biobehavioral Sciences, Teachers College – Columbia University, New York, NY, United States; ^3^ Department of Kinesiology and Physical Education, Northern Illinois University, Dekalb, IL, United States; ^4^ Department of Exercise and Rehabilitation Sciences, Merrimack College, North Andover, MA, United States; ^5^ Center for Cognitive Neuroscience, Duke University, Durham, NC, United States; ^6^ Yale Stress Center, Yale School of Medicine, New Haven, CT, United States; ^7^ Department of Kinesiology and Health Education, The University of Texas at Austin, Austin, TX, United States; ^8^ Faculty of Physical Activity and Sports Sciences, Universidad de León, León, Spain; ^9^ Department of Psychology, Southern Connecticut State University, New Haven, CT, United States; ^10^ Connecticut Community Colleges Nursing Program, Gateway Community College, New Haven, CT, United States; ^11^ Center for Medical Informatics, Yale School of Medicine, New Haven, CT, United States; ^12^ Department of Psychiatry and Behavioral Sciences, University of Minnesota, Minneapolis, MN, United States; ^13^ Center for Pain, Research, Informatics, Medical Comorbidities and Education Center (PRIME), VA Connecticut Healthcare System, West Haven, CT, United States

**Keywords:** motivation, motivation states, desires, physical activity, exercise, qualitative study, focus groups, stress

## Abstract

Motivation for physical activity and sedentary behaviors (e.g., desires, urges, wants, cravings) varies from moment to moment. According to the WANT model, these motivation states may be affectively-charged (e.g., felt as tension), particularly after periods of maximal exercise or extended rest. The purpose of this study was to examine postulates of the WANT model utilizing a mixed-methods approach. We hypothesized that: (1) qualitative evidence would emerge from interviews to support this model, and (2) motivation states would quantitatively change over the course of an interview period. Seventeen undergraduate students (mean age = 18.6y, 13 women) engaged in focus groups where 12 structured questions were presented. Participants completed the “right now” version of the CRAVE scale before and after interviews. Qualitative data were analyzed with content analysis. A total of 410 unique lower-order themes were classified and grouped into 43 higher order themes (HOTs). From HOTs, six super higher order themes (SHOTs) were designated: (1) wants and aversions, (2) change and stability, (3) autonomy and automaticity, (4) objectives and impulses, (5) restraining and propelling forces, and (6) stress and boredom. Participants stated that they experienced desires to move and rest, including during the interview, but these states changed rapidly and varied both randomly as well as systematically across periods of minutes to months. Some also described a total absence of desire or even aversion to move and rest. Of note, strong urges and cravings for movement, typically from conditions of deprivation (e.g., sudden withdrawal from exercise training) were associated with physical and mental manifestations, such as fidgeting and feeling restless. Urges were often consummated with behavior (e.g., exercise sessions, naps), which commonly resulted in satiation and subsequent drop in desire. Importantly, stress was frequently described as both an inhibitor and instigator of motivation states. CRAVE-Move increased pre-to-post interviews (*p* < .01). CRAVE-Rest demonstrated a trend to decline (*p* = .057). Overall, qualitative and quantitative data largely corroborated postulates of the WANT model, demonstrating that people experience wants and cravings to move and rest, and that these states appear to fluctuate significantly, especially in the context of stress, boredom, satiety, and deprivation.

## Introduction

Physical inactivity and sedentarism plague the United States and other developed countries. Sitting time, not including other sedentary behaviors like napping, has steadily increased among Americans to nearly 5.9 h/day (1), and only 24% of American adults meet the physical activity guidelines for combined aerobic and strength training (2). Structured exercise (e.g., a 30-minute run) is just one facet of energy expenditure (EE). Other sources of EE include non-exercise activity thermogenesis (NEAT). This includes lifestyle physical activity (PA; e.g., walking to a train station) and spontaneous physical activity (SPA; e.g., standing up, getting a glass of water, fidgeting, etc.) (3). Active and sedentary behaviors vary widely across the day and between days, are not necessarily synchronous and, in fact, they can be demonstrated simultaneously (4–7). Unfortunately, current models of health behavior are insufficient to explain and predict the complexity of human movement and EE as they focus on habitual activity and trait-like motives without consideration for variations in movement and motivation from moment to moment (8–11). There has also been criticism that decades of research focused on cognitive aspects of physical activity behavior have overshadowed constructs of emotion and motivation, despite the low predictability of such factors (12, 13). Improvements have recently been made in modelling physically active behaviors, as with the Affective-Reflective Theory (ART) of physical inactivity and exercise (14), the dual process model from Conroy and Berry (15), and the Affective Health Behavior Framework (AHBF) (16). These theories incorporate the influences of affect, cognitive deliberation, hedonic motivation, and the idea of a final action impulse—a motivational catalyst or endpoint that instigates both active and sedentary behaviors.

In addition, common to these and other models is the idea of subjective wanting or desiring to move and rest, also known as motivation states (17). For instance, there are times when people may want to get up and stretch their legs, exercise, or go for a walk. Likewise, they may desire to sit on the couch, take a nap, or lay down in bed. In this case, “desire” and “want” are used interchangeably, as has been done by other researchers (18), but they can also be used separately to denote influence from reflective or appetitive systems (12). These motivation states may be experienced as strong urges and cravings, conspicuously incorporating the idea of felt tension and may be experienced as positive or negative. Collectively, desires, wants, urges, and cravings are known as affectively-charged motivation states (ACMS) (19). These occur in both healthy individuals, where they may often go unnoticed, and also in clinical populations, where they can be quite bothersome and even disabling (20). The basis of these states could be a basic drive to move and be active (21), which initially Feige (22) and more recently others (17) recognized as the foundation of physical activity motivation. The recognition of ACMS could significantly enhance our theoretical models as they: (1) apply to any rewarding behavior, (2) can change from moment to moment, and (3) incorporate aspects of affective response (16, 23). Over the last few years, Stults-Kolehmainen and colleagues (17, 20, 21, 24–27) have developed the idea of motivation states for movement and rest in one of the first efforts to incorporate these ideas into behavioral models.

To understand how desires and urges for movement and sedentarism interact, Stults-Kolehmainen and colleagues (17) developed the WANT model (Wants and Aversions for Neuromuscular Tasks). This heuristic is a circumplex-type framework that incorporates three main factors (i.e., move and rest; want and lack of want; approach and withdrawal). A complete set of postulates of the WANT model include:

1. Humans have reflective and appetitive desires to move and rest.
2. Desires for movement and rest are characterized as two separate systems, and not opposite sides of the same axis.
3. There is both approach and avoidance motivation for movement and rest (e.g., one might be actively dis-wanting to move) (8, 23, 28).
4. These desires vary in strength or intensity (29) from very weak to nearly unavoidable/maximal, where they might be felt as an urge or craving.
5. Wants/desires are highly transitory psychological states.
6. They change in response to behavior (i.e., the provision, deprivation, or avoidance of certain physical stimuli, such as exercise).
7. They interact asynchronously (e.g., one may be high in both, low in both, or anywhere in-between).
8. There may also be a total lack of desire, as in meditative or sleeping states, or perhaps total apathy or indifference.
9. They differ from psychosomatic sensations, such as energy and fatigue.
10. They differ from emotions; however, the experience of desire for movement and rest might vary systematically with certain emotions (e.g., stress responses, fight, flight, fright, freeze), situations (e.g., sporting event, sudden terror) and conditions (e.g., illness) (23).

The WANT model is influenced by theories mentioned above, but perhaps most concordant with the concept of motivation control systems from Frijda and colleagues (30–32), who articulated ideas of motivation states, strength of urges, wanting vs. not wanting, approach vs. withdraw, a center point of no desire (e.g., apathy, disinterest, indifference), and how these relate to emotion. Also related is the Elaborated Process Model of self-regulation by Inzlicht and colleagues (33), who describe opposing motivational systems of “exploration, leisure & want-to” vs. “exploitation, labor & have-to.” Some researchers, however, have presented data and models that are less supportive of our model. These have speculated that: (1) desires to move and be active have weak influences on physically active behaviors, (2) desire to move may be subservient to desires to rest and be sedentary, (3) avoidance motivation (e.g., dread of movement) rather than approach or want of movement, is most influential or (4) desires to be active may not exist at all (12, 16, 29, 34–37). Importantly, there appears to be a consistent logical fallacy from many of these sources and others—that low exercise behavior and large waist lines observed across the population are evidence that most people do not want to move (38, 39). Nevertheless, our recent work seems to dispute these assertions (24).

Our laboratory recently conducted a series of studies (24) to provide initial validation for the concept of affectively-charged motivation states (ACMS) for physical activity and sedentarism and the WANT model. With 846 participants, we developed a tool to measure ACMS, called the CRAVE (Cravings for Rest and Volitional Energy Expenditure), and subsequently conducted factor analyses to analyze both “right now” and “past week” versions. One hundred and twenty-seven people from New England were then tracked over a two-year period, where it was determined that ACMS have properties more similar to states than traits. In a later study, 21 undergraduate students from Texas completed the CRAVE before and after a maximal treadmill test, where it was found that motivation states to move declined precipitously (Cohen's d_av_ = 1.05) and to rest increased (Cohen's d_av_ = 0.82). In a separate study, 41 students from the American Midwest were measured 3 times across a lecture period, where it was found that desires to move increased 20% just before class dismissal, while desires to rest decreased 17%. In this last investigation, ACMS were moderately related to sensations of energy and fatigue. In line with expectations, these studies verified that motivation states are predicted by preceding behaviors. Overall, we can conclude that the concept of motivation states to be active and rest is valid and worthy of further exploration.

Despite the initial progress in developing and validating the concept of motivation states for physical activity and rest, many challenges need to be faced. First, there is still a dearth of evidence in the area, as noted by influential scientists in the area of exercise psychology (12). Second, the concept is still largely theoretical and lacks ecological validation—the voice of opinion from non-scientists. That is to say, in investigations up until this time, the concept has been largely limited to responses on an instrument in controlled settings, without greater naturalistic context. For instance, the way people describe motivation states in common language may not include the terms “desire”, “want”, “urge” or “craving”. Similarly, the WANT model needs further development and ecological validation as it may be missing important postulates that could be identified qualitatively. Conversely, important suppositions in the model (e.g., two axes, magnitude, approach vs. withdrawal) may lack sufficient ecological validity. Further development is also needed as the WANT model is largely descriptive and explanatory without being predictive. In this regard, 1) there is little evidence to show a strong connection between ACMS and future behavior (26), and 2) there are currently no adequate predictive models that incorporate desires and wants to move and rest. Qualitative research can fill that gap, using insights from participants to identify mechanisms for theory and conceptual model development (40).

Consequently, to further develop and validate the concept of affectively-charged motivation states (ACMS) and the WANT model, there are five aims of the current investigation.

1. To extend the quantitative validation of: (a) the CRAVE scale, (b) the ACMS concept (i.e., that people do have wants and desires), and (c) the WANT model.
2. To further validate qualitatively the ACMS concept for movement and rest; to uncover if respondents recognize these states in their own personal experience and how they might be described in layman's terms.
3. To further validate qualitatively postulates of the WANT model.
4. To understand if ACMS relate to and spur physically active and sedentary behavior.
5. To generate information and themes to further develop the concept of motivation states and the WANT model and/or develop stronger predictor models of behavior.

Aims 2–4 will use a qualitative deductive approach and aim 5 will use a qualitative inductive approach.

Regarding the deductive analyses, we hypothesized that:

1) motivation states (assessment *via* the CRAVE scale) would change over the course of an interview period (pre to post),
2) qualitative evidence would emerge from interviews to support: (a) the ACMS concept and (b) postulates of the model,
3) qualitative evidence that ACMS are linked to future physically active and sedentary behavior.

## Materials and methods

### Experimental approach

To address the aims and hypotheses of this study, we chose a mixed methods approach combining qualitative and quantitative methods. Participants were interviewed in focus groups (described below) with quantitative measures collected before and after the interviews.

### Participants

Participants were 17 college undergraduate students (mean ± SD: age = 18.6 ± 0.94y; BMI = 26.1 ± 6.5; 7 people of color; 12 first-year students) enrolled in the Honors Program at the university. We queried about gender and not biological sex. There were 13 women, 2 men, and 2 individuals identifying as non-binary. Participants were largely recruited in-person during classes by word of mouth with a script by one of the principal investigators (TG). Participants received a $30 gift card for participation.

### Procedure

The interviews took place in-person, in a private setting on the university campus, in one of seven focus groups that incorporated one to four participants at a time. Before commencement of the interviews, participants were briefed on the study purpose—to better understand the determinants of movement behaviors in humans, such as the urge to be active. Procedures, potential risks, and requirements for participation were discussed with all participants. They completed a consent form indicating their willingness to participate and have the interview digitally recorded. Upon completion of the informed consent participants filled out a short demographic questionnaire and CRAVE questionnaires (Past Week and Right now versions). Following the completion of these questionnaires, participants engaged in a focus group interview that presented 12 structured questions (Supplementary Data Sheet S1). A researcher with extensive experience in qualitative research (TG) conducted the focus groups. Finally, participants ended by completing the CRAVE (Right now version) questionnaire one last time. Interviews were recorded by the interviewer and transcribed by a professional scribe.

### Interview questions

Questions were structured to be balanced between move and rest (i.e., 4 specific to activity, 4 specific to rest, 4 for both move and rest). The first 4 questions regarded the validation of the concept and model. Questions 5–12 were created with the idea of conceptual and model development. Questions were always presented in the same order, with questions 1 and 2 intended to prime participants for later questions. The interview responses were free flowing in that the same person did not always respond first. Once the interviewer finished posing the question, the first person who wished to comment was allowed to do so; however, each person had a chance to respond to every question. Participants typically engaged in a discussion format regarding their feelings, perceptions and observations related to the topics at hand. When necessary, and to facilitate greater discussion, probes were used by the researcher to elicit more detailed responses.

### Quantitative measure

CRAVE (Cravings for Rest and Volitional Energy Expenditure): The CRAVE is a 13-item questionnaire with two versions, "past week" and "right now", which has been validated across six studies (24, 26), demonstrating excellent psychometric properties. For this study, the past week version was used just at the beginning and the right now version was used both pre and post. Six scale items relate to physical activity (e.g., “move my body”), and 7 items are related to sedentary behaviors (e.g., “do nothing active”). In validation testing, an exploratory structural equation model (ESEM) revealed that 10 items should be retained, loading onto two factors (5 each for Move and Rest). Consequently, the remaining 3 items are unscored fillers. Move and Rest factors are correlated moderately and inversely (*r* = −.71 and −.78, in two different studies). Reliability of the scale in the same studies, as determined by McDonald's *ω*, was very high (both .97). The CRAVE has good test-retest reliability and reliably measures state-like properties of motivation. Across-session interclass correlations (ICC) for Move (ICC = 0.72–0.95) and Rest (ICC = 0.69–0.88) are higher than those measured across 24-months (Move: ICC = 0.53; Rest: ICC = 0.49). The CRAVE is sensitive to changes with exercise testing, with Move decreasing with a maximal stress test (Cohen's d_av_ = 1.05) and Rest increasing (Cohen's d_av_ = 0.82). It has small to moderate associations with sensations of energy, fatigue, tiredness, and deactivation.

### Data analysis

Quantitative data was analyzed with paired t-tests with the Jamovi statistical package (Version 2.2) (41). For qualitative data, researchers used content analysis as described by Hsieh & Shannon (42) and formerly utilized by one of the first authors (43) to analyze results. A deductive approach was used for theoretical validation—to identify support or disagreement with both: (A) the concept of ACMS for movement and rest and (B) the WANT Model. An inductive approach was used for concept development. These approaches were conducted simultaneously for efficiency. Two analysts, both experts in the content area (TG and MSK), started by identifying lower order themes, which were entered into Microsoft Excel. Associated data from interviewees was tagged to lower order themes (LOTs). For the inductive approach, analysts independently inspected LOTs to generate higher order themes (HOTs). Every LOT was tagged to a HOT. Later, HOTs were sorted into a reduced number of bins to create super-higher order themes (SHOTs). In creating SHOTs, additional theory was considered, such as the Elaborated Process Model of self-regulation (33), motivation control systems (30–32), Self-Determination Theory (44), the Incentive Sensitization Model (ISM) of rewarding behaviors (45, 46), and the Theory of Hedonic Motivation (12). In the case of disagreement in the creation of HOTs and SHOTs, a third author (NSB) provided the tiebreaker.

## Results

### Quantitative analysis

CRAVE-Move was rated higher than CRAVE-Rest for both pre- (*p* = .022, Cohen's d = 0.61) and post-interviews (*p* <  .001, Cohen's d = 1.48). PW and RN versions of CRAVE-Move were moderately correlated (*r* = .51, *p* < .05). Respondents rated their CRAVE-Move as being higher “over the past week” (PW) than “right now” (RN) (33.7 ± 8.0 vs. 28.9 ± 9.8). PW and RN versions of CRAVE-Rest were also moderately associated (*r* = .49, *p* < .05). We could not reject the null hypothesis that there was no difference for rest “over the past week” vs. “right now” (16.5 ± 7.6 vs. 17.3 ± 10.9). See Table 1.

**Table 1:** Correlation Matrix (Pearson's *r*) and descriptive statistics for “Past week” (PW) and “Right now” (RN) versions of the CRAVE Scale, measuring desires and wants to move and rest.

	RN MOVE PRE	RN REST PRE	RN MOVE POST	RN REST POST	PW MOVE PRE	PW REST PRE
RN MOVE PRE	—					
RN REST PRE	–.66**	—				
RN MOVE POST	.59*	–.08	—			
RN REST POST	–.45	.39	–.71**	—		
PW MOVE PRE	.51*	–.53*	.42	–.58*	—	
PW REST PRE	–.35	.49*	–.42	.67**	–.74***	—
Mean	28.9	17.3	35.3	11.9	33.7	16.5
SD	9.8	10.9	8.9	8.3	8.0	7.6

* *p* < .05, **p < .01, ***p < .001.

**Figure 1:**
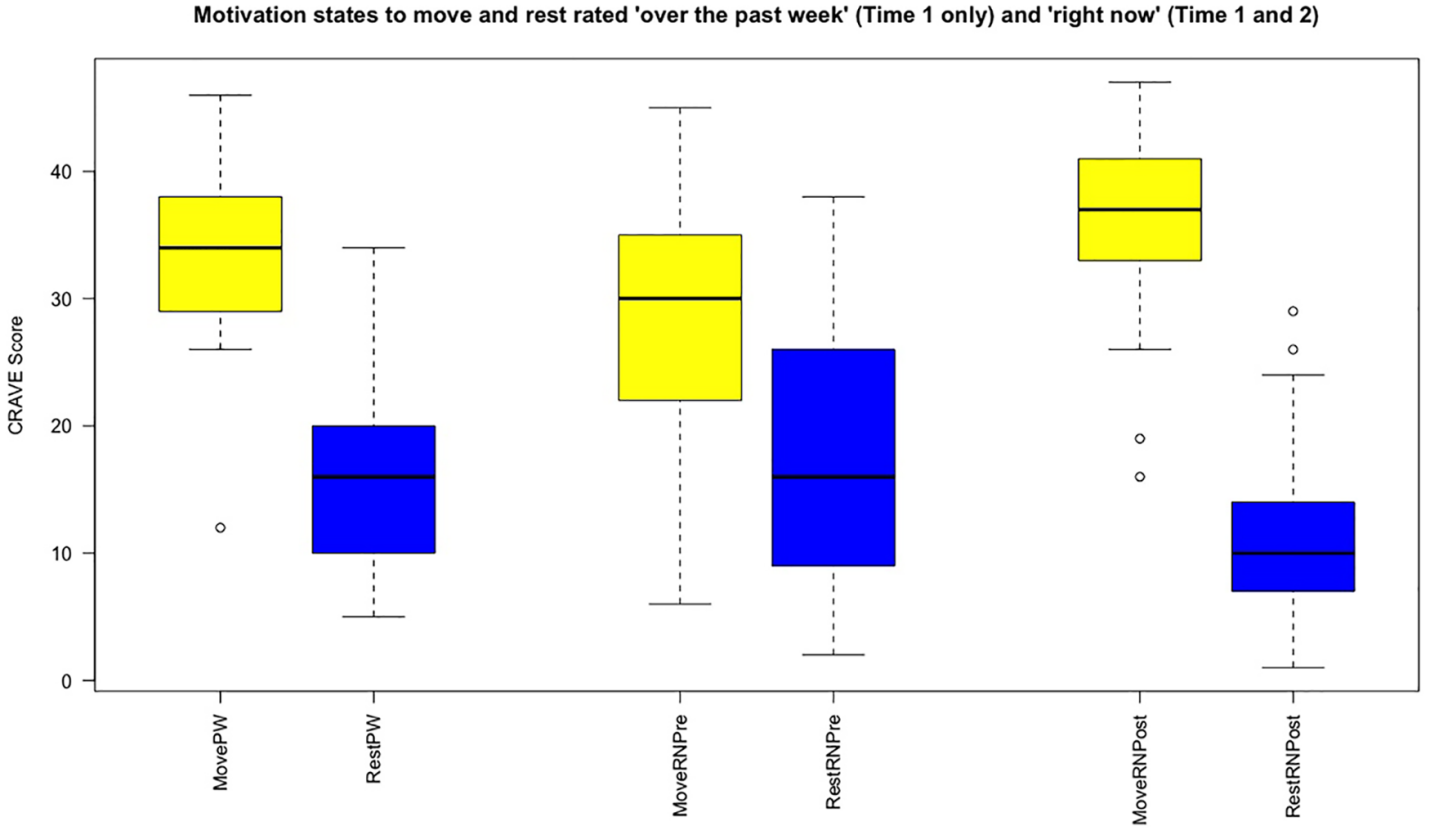
Motivation states to move and rest rated “over the past week” (PW, pre only) and “right now” (RN, pre and post).

CRAVE-Move (right now) significantly increased across the interviews from 28.9 (SD = 9.8) to 35.3 (8.9) (*p* = .006, Cohen's d = 0.76). CRAVE-Rest demonstrated a trend to decline: 17.3 (SD = 10.9) to 11.9 (8.3) (*p* = .057, Cohen's d = 0.50). Variance decreased meaningfully, as seen in Table 2 and Figure 1.

**Table 2:** Paired samples T-tests comparing pre- versus post-interview time points for both “Right now” (RN) and “Past week” (PW) versions of the CRAVE scale.

								95% Confidence Interval	
		t	df	*p*-value	Mean difference	SE difference	Effect Size (Cohen's d)	Lower	Upper
RN MOVE PRE	RN MOVE POST	−3.129	16	.0065	−6.412	2.049	−0.759	−1.292	−0.208
RN REST PRE	RN REST POST	2.054	16	.0567	5.412	2.635	0.498	−0.014	0.997
RN MOVE PRE	RN REST PRE	2.533	16	.0221	11.588	4.575	0.614	0.086	1.127
RN MOVE POST	RN REST POST	6.091	16	<.0001	23.412	3.843	1.477	0.771	2.162
PW MOVE PRE	RN MOVE PRE	2.234	16	.0401	4.824	2.160	0.542	0.024	1.045
PW REST PRE	RN REST PRE	−0.322	16	.7517	−0.765	2.376	−0.078	−0.553	0.399
PW MOVE PRE	PW REST PRE	4.874	16	.0002	17.176	3.524	1.182	0.546	1.797

### Deductive qualitative analyses

#### Evidence for motivation states

Thematic findings from these qualitative interviews, specifically Questions 3, 4 and 12 (presented later), corroborated the concept of ACMS for movement and rest. In question 3 (“Do you want to move right now?”), 12 reported “Yes” and indicated some desire to move; five indicated “No”. Of these, some respondents qualified their answer by noting they only wanted to move at a low intensity (*n* = 2), and one “No” was emphatic. In Question 4, 12 respondents reported that they did not want to rest; 4 responded they wanted to rest, and 1 did not know. Of these, 1 person noticed a conflict between wanting to move and rest. Two respondents noted they wanted to rest “a lot”, and 1 respondent noted she/he did not want to rest “at all”.

Throughout the focus group sessions, participants remarked frequently about their desires to move, be active, but also to rest, both over the past week (e.g., “I have been wanting to move around a lot”) and right now (during the interview, e.g., “I want to get out and train”). Interviewees also provided evidence of aversions or avoidance of both movement and rest. While many of these statements were unambiguous, others were suggestive and less concrete, [“I am feeling like I wish we were doing a bit more (exercise)”]. Table 3 provides a compilation of statements supportive of the concept of affectively-charged motivation states.

**Table 3:** Qualitative evidence for ACMS for movement/physical activity/exercise as well as rest/sedentary behaviors.^a^

		MOVE	REST
WANT	Recently	“My desire has been pretty high to move and be active.” (9/17, Participant A) “I have been wanting to move around a lot.” (9/17, B) “I am trying to get [my desire] back up so I can actually work out.” (9/21, A) “Over the past week I wanted to move a little bit more.” (9/21, A) “In the past few days I've wanted to move around a little bit more.” (9/21, A) “I think I am still on a ‘move thing’ right now. I went hiking on Saturday, and afterwards I went out [to socialize]. Even though I am tired I don't really want to stop.” (9/21, C) “I really wanted to move over the past week—more than I have consistently over the first few weeks of the semester.” (9/10, B) “I get random bursts of energy, and it makes me really motivated, and it makes me want to get more done, and it makes me wanna go on runs, go on walks, get more homework done, or get the next week's homework done.” (9/13, A) “… Over spring break our coach tells us … ‘You shouldn't be training … You shouldn't be pushing yourself to any degree that's beyond something casual’ … By the time you get back from spring break you’re dying to get a hard workout in [and] get that sweat going … You miss it, and … you’re reinvigorated [and] wanting to train and push yourself. It's a craving.” (9/17, A) “The workload is not too heavy—so I wish we were doing a bit more [exercise].” (9/17, A)	“Over the past week, I have wanted to rest a lot.” (9/10, A) “I [have] just wanted to sit down.” (8/31, A) “I [have] just wanted to go to sleep.” (9/17, A)
	Now	“I have had a jam-packed day full of stuff, and I am going to be going until 11pm—so it's a bit more like jolty, anxiety movement. I want to kick my foot around a little, or shake a bit, just to get rid of that nervous energy, in terms of moving, in terms of exercise or working out”. (9/27, A) “… I want to work out.” (8/31, C) “I wanna get out and train”. (9/17, A) “I’d say yeah, I wanna move right now, but not like I’m itching to get out of my seat and go run.” (9/17, A) “ … I feel urged to move and get stuff done …” (9/27, A) “I want to [move] but I guess I don't really want to heavily exert myself …” (8/31, D)	“I definitely crave rest a lot. I crave just sitting! Sitting is nice. It is very good. Sitting here during the interview is very good. It's nice.” (9/27, A) “I would really love to be laying down right now in a fetal position with my teddy bear. That would be ideal because I don't get enough sleep, and it would be nice to just take a nap right now.” (9/27, A)
DON'T WANT	Recently	“I have NOT wanted to move more than the necessary amount.” (9/21, A) “A time where I really wanted to just slow down and do nothing or just rest … was, not really rest, in itself, but just not move.” (9/10, B) “… We’ll finish a game”. [Maybe] the next day we have off. I’m like, “Oh, I wanna go to the gym”. He's like, “I’m wiped out from yesterday. I don't wanna go to the gym”. (9/17, A)	“Last night I felt like I couldn't fall asleep. I was just awake and had this jitteriness—almost where it was hard for me to fall asleep. I didn't want to rest.” (9/17, A) “I have not been willing to get any rest, [and] because of that, it has been impacting my sleep.” (9/21, B)
	Now	“I don't want to move at all.” (9/10, B)	“I just do NOT want to get any rest.” (9/21, B)

^a^ Note that some participants made slightly conflicting statements from one part of the interview to another, or more simply, their motivation state changed over time.

There was also some doubt about the desire or want to move. For instance, one respondent said,

“… When it comes to ‘urge’ and ‘crave’ it's a natural thing [where] you crave sleep because you can't really just stay up all the time—because you need to sleep. It's more primal, I guess. Because everyone has to sleep. You don't have to move. Well, I guess it depends. There are people who don't really move, but there are some people that do. But everyone sleeps, no matter how active you are.” (9/15/21, Participant A).

There was even some doubt about the desire or urge to rest, “I don't see an urgency necessarily to rest, because I gotta reach that brink of exhaustion, to feel that I have earned the right to rest. … I don't feel an urgency to rest.” (9/27, A). Also, “Do I want to rest physically? I don't know, but I am ok with being active because I feel like my brain needs a rest” (8/31, D).

#### Changes in motivation states from pre- to post-interview

Question 12 revealed that at the end of the interview, 12 participants declared a greater desire to move, and two had no perceived changes in desires to move. Of the three remaining, they reported increased awareness of affectively-charged desires for movement and rest behaviors, which was corroborated by two other participants. One participant noted how this awareness also related to behavior,

“I think I get urges to move because I always have a twitch going on, and I'll move my legs a lot, like I am doing now. They are always moving, and if I notice it happening more, I feel like, ‘Okay, I need to get up and walk around’, even if it's just while I am listening to a class online. … I have to do something!” (9/21, C).

One of the participants who reported no change in movement desires contrasted that with a report of a decrease in rest. Another respondent reported, “Interestingly enough, I think I’ve actually woken up in the hour that I’ve been here …” (9/17, A).

#### Support for the WANT model

Respondents' comments provided supporting evidence for all postulates of the WANT model. One postulate, “Desires to move and rest interact asynchronously (e.g., one may be high in both or low in both or anywhere in-between),” had few supporting statements. However, in regard to this tenet, a respondent reflecting on a stressful situation noted,

“I was a bit hungover, and I was stuck to my bed because I was a bit nauseous, but I [couldn't] fall asleep. [I thought] ‘If you can't rest, you should be doing something’, and it was very annoying because I wanted to begin cleaning my room. ‘I’m awake, I should be moving’, but I needed my eyes to be closed and a pillow over my head. I couldn't satisfy the urge to move and get stuff done, and that was very stressful.” (9/27, A).

There was some evidence against specific postulates of the WANT model. For instance, concerning the supposition that “Desires vary in strength from very weak to nearly unavoidable/maximal,” an interviewee remarked,

“I would say that want, desire, and urge—the whole set—feels the same to me. I don't think that they are super different.” (8/31, B).

See Table 4.

**Table 4:** Qualitative evidence for the WANT model.

#	Postulate of the WANT model	Qualitative evidence (for)	Qualitative evidence (against)
1	Humans have desires to move and rest.	See Table 3 above.	
2	Desires for movement and rest are two separate systems.	“If I just don't want to go to practice [for sports]—I want to rest that day. That's completely different from craving and needing to rest.” (8/31, Participant A)	
3	These desires have both approach and avoidance motivation.	See Table 3 above.	
4	Desires vary in strength from very weak to nearly unavoidable/maximal, where they might be felt as an urge or craving.	“… ‘want/desire’ are a little lower compared to ‘urge’ and ‘crave’. Those are more towards the need to do something. When you want to do it, you don't necessarily do it, but if you have the urge, or if you really crave to do it, then you are going to do it …” (8/31, A) “In terms of wanting to move … desires are where it would be nice if I moved—it would be nice if I worked out, but it's never going to happen.” (9/27, A) “I think that cravings or urges to rest are BOTH physical [sensations] and mental [thoughts]. However, when I want to rest—I feel that ‘want’ is usually either physical or mental, but not both. For ‘want’, it's like, ‘Oh, I'm kinda tired; I want to rest’, but I still have the [physical] energy in me to keep doing something. I feel that ‘crave’ [to rest] is when everything in me is just like, ‘I can't do this anymore; I just need to stop.’ (8/31, D)	“I would say that the want, desire, and urge—the whole set—feels the same to me. I don't think that they are super different.” (8/31, B)
5	Wants/desires are highly transitory—representing a psychological state.	“I'd say a ‘want’ to rest is maybe more of a short-term feeling for me. I just finished a game, you know, my body's tired. I just want to chill out for a second, rehydrate, eat something. Whereas ‘desire’ or ‘urge’ to rest, I feel is more created by a longer-term circumstance, whether it's that we've been in pre-season now, and you're training twice a day, every day, and you're just thinking, ‘All I want is to just relax and rest and catch up on sleep’, or whatever it may be.” (9/17, A) “I think that ‘want’ and ‘desire’ is more like a superficial thing. It's not going to last. It's short term, but then ‘urge’ or ‘crave’ is almost like you physically need to.” (9/17, C) “I only crave rest right after I wake up, because I feel that as soon as I get going in the day, its fine. If I actually get myself up, the craving for rest goes away. So, I will wake up and it's, ‘Oh my god—its 7am. All I want is to go back to the bed.’ And then as soon as I go brush my teeth or something, I'm thinking, ‘What was I tired for?’, and it's fine. I woke up that way essentially, and the craving goes away, and I am fine for the rest of the day.” (9/21, C)	
6	ACMS change based on previous behaviors (i.e., the provision or avoidance of certain physical stimuli, such as exercise).	“‘Craving’ is more when I am doing something [highly] repetitive because I am bored of the same activity, so I want to do something else, if that's resting, being on my phone, or just laying down, or watching TV. While ‘want’ and ‘desire’ is when I am doing something in the moment—let's say I am working out, and I think, ‘Oh, I want to stop’”. (8/31, B) “… Over spring break our coach tells us, ‘You shouldn't be training … You shouldn't be pushing yourself to any degree that's beyond something casual’ … By the time you get back from spring break, you’re dying to get a hard workout in that gets the sweat going … You miss it, and … You’re reinvigorated [and] wanting to train and push yourself. It's a craving.” (9/17, A) “I think I have the urge when something is going on in my life where I just need to get out, and I need to run if I have been sitting for a long time. I need to just run on vacations. We would always stop at a rest area for a road trip, and I would literally just get out[!]—because when I was little I'd just run to the playground. I needed to run because I craved moving, because I was in the car for about 10 h.” (8/31, D) “… after a long time of movement you want to rest, but [after] a long time of resting, you DON'T want to get up and, you know, run two miles.” (9/8, A)	
7	Desires to move and rest interact asynchronously.	“I was a bit hungover, and I was stuck to my bed because I was a bit nauseous, but I [couldn't] fall asleep. [I thought] ‘If you can't rest, you should be doing something’, and it was very annoying because I wanted to begin cleaning my room. ‘I’m awake, I should be moving’, but I needed my eyes to be closed and a pillow over my head. I couldn't satisfy the urge to move and get stuff done, and that was very stressful.” (9/27, A)	
8	A total lack of desire is possible	“I don't want to do anything right now.” (9/21, D)	
9	ACMS differ from psychosomatic sensations, such as energy and fatigue.	“I get random bursts of energy, and it makes me really motivated, and it makes me want to get more done, and it makes me wanna go on runs, go on walks, get more homework done, or get the next week's homework done.” (9/13, A) “The desire to rest is more motivated by my body and how my body is feeling, and the desire to move is more like a mental thing.” (9/17, A) “I am just too tired. I have wanted to be active, but I just don't always have that energy.” (9/10, A)	
10	They differ from emotions but might vary systematically with certain emotions and situations.	“What makes me want to move is just the joy I get from playing sports. I enjoy exercising [and] definitely feel motivated …” (9/17, A) “When I get overwhelmed, I prefer to rest and just be alone resting.” (9/10, C)	

#### Impact of affectively-charged motivation states (ACMS) on subsequent behavior

We found qualitative evidence that motivation states were related to aspects of subsequent movement and sedentary behavior—in type, quantity and in quality of motor behaviors. The effect on behavior was often related to the strength of the ACMS. One participant stated, “If I really want to exercise, I will make time for it.” (9/10, B). Respondents also reported that motivation states did not result in behavior enactment/consummation. For instance,

“‘Want’ is more knowing I should, but it doesn't incite me to actually do it. Want is just, ‘I should probably do this, because I know it's good for me’, but I don't actually do it.” (8/31, D).

See Table 5.

**Table 5:** Qualitative evidence that affectively-charged motivation states (ACMS) have influence on movement and sedentary behaviors.

	Qualitative evidence (for)	Qualitative evidence (against)
Movement/physical activity/exercise	“If I really want to exercise, I will make time for it.” (9/10, Participant B) “The ‘want’ and ‘desire’ are feeling motivated, but not really motivated, and then [‘urges’ and ‘cravings’] are, “Oh, I'm going to do this. I'm going to get up. I'm going to move. You want to get out. You want to do the exercise or whatever the movement is.” (8/31, C) “I remember all of last summer, every morning, I craved to work out, and I craved to practice even though I couldn't go. In my own time in my backyard I would work out and mimic a practice by myself because I craved it.” (8/31, B)	“‘Want’ is more knowing I should, but it doesn't incite me to actually do it. ‘Want’ is just, ‘I should probably do this, because I know it's good for me,’ but I don't actually do it.” (8/31, D) “I sometimes actually move when I have a ‘want’ to move, but it takes a lot more willpower to do it.” (8/31, D) “I have a lot of friends on social media who will post gym selfies, and when I see those I'm feeling like, ‘Ah, look at them. I should probably do that!’ That's an outside factor that potentially pushes me to want to move or do what they are doing. It never really happens, but definitely I mentally get that, but not physically.” (9/27, A)
Rest/sedentary behaviors	“… When I desire rest, it's much more appealing [than movement]. And I very much try my very best to make it happen. And if I desire to take a nap, I feel you will be able to tell it more. I'll be kinda drooping a bit. I'll be a bit more tired- not as talkative. Versus if I want to move, I don't know if you'd necessarily see that in a physical appearance.” (9/27, A) “I feel that ‘crave’ [to rest] is when everything in me is just like, ‘I can't do this anymore; I just need to stop.’” (8/31, D) “… It's a natural thing [where] you crave sleep, because you can't really just stay up all the time, because you need to sleep. It's more primal, I guess. Because everyone has to sleep.” (9/15, A)	None observed

### Qualitative inductive analyses

#### Lower-Order theme (LOT) identification

Investigators found 435 lower-order themes (e.g., “move for sport”, “rest and be lazy”), only 25 of which were identical between raters, resulting in 410 unique lower order themes. LOTs generated per interview question ranged from 16 for Question 12 to 65 for Question 7 (mean = 36.3, SD = 12.4). There was a total of 753 counts (e.g., instances or tags) across all LOTs. Counts (e.g., instances or tags) per LOT identified were 1.7 (range 1.1 to 3.0). In the first eight questions, move queries resulted in 180 LOTs, and rest resulted in 145, but total counts from move queries were 285 and from rest were 264. Overall, these data demonstrate that many lower order themes were identified (for both move and rest factors). LOTS were tagged to participants' comments.

#### Higher-Order theme (HOT) identification

The two analysts generated the same (or highly similar) higher-order themes only 22.4% of the time. Discordant HOTs were sent to analyst 3, who chose analyst 1's HOT in 33.6% of instances. In 1.3% of instances, the analyst was unable to make a determination, resulting in the items being discussed until consensus. In the end, 43 higher order themes were agreed upon.

The 10 most common HOTs (based on frequency of LOTs in ach HOT) were: (1) “sensations/stimulation” (*n* = 62 LOTs), (2) “demands” (*n* = 52), (3) “facilitators of movement” (*n* = 40), (4) (tie) theoretical support (*n* = 37), (4) (tie) “physical sensations” (*n* = 37), (6) “cycles/variation” (*n* = 35), (7) (tie) “stress” (*n* = 32), (7) (tie) “exhaustion threshold” (*n* = 32), (7) (tie) “deprivation/satiation” (*n* = 32), and (10) “barriers for movement” (*n* = 31).

#### Super higher-order theme (SHOT) identification

From the 43 HOTs, super higher order themes (SHOTs) were created. Analyst 1 sorted the 43 themes in 10 clusters, which included: (1) Stable change / biorhythms, (2) Factors affecting change in motivation states, (3) Processes of control, (4) Impulse control/Impulsivity, (5) Objective-oriented, (6) Moderators, (7) Sensations, (8) Strength of motivation states, (9) Theoretical postulations, and (10) Stress factors. Analyst 2 sorted the HOTs into three SHOTs: (1) Theoretical support, (2) Behavioral processes, and (3) Stress. Through a process of consensus, six were designated: (1) “People experience movement urges”, (2) “Change”, (3) “Autonomy”, (4) “Objective-orientation”, (5) “Moderators”, and (6) “Stress effects”. These were then presented collectively at an international conference (see Acknowledgements), and feedback was garnered.

**Figure 2:**
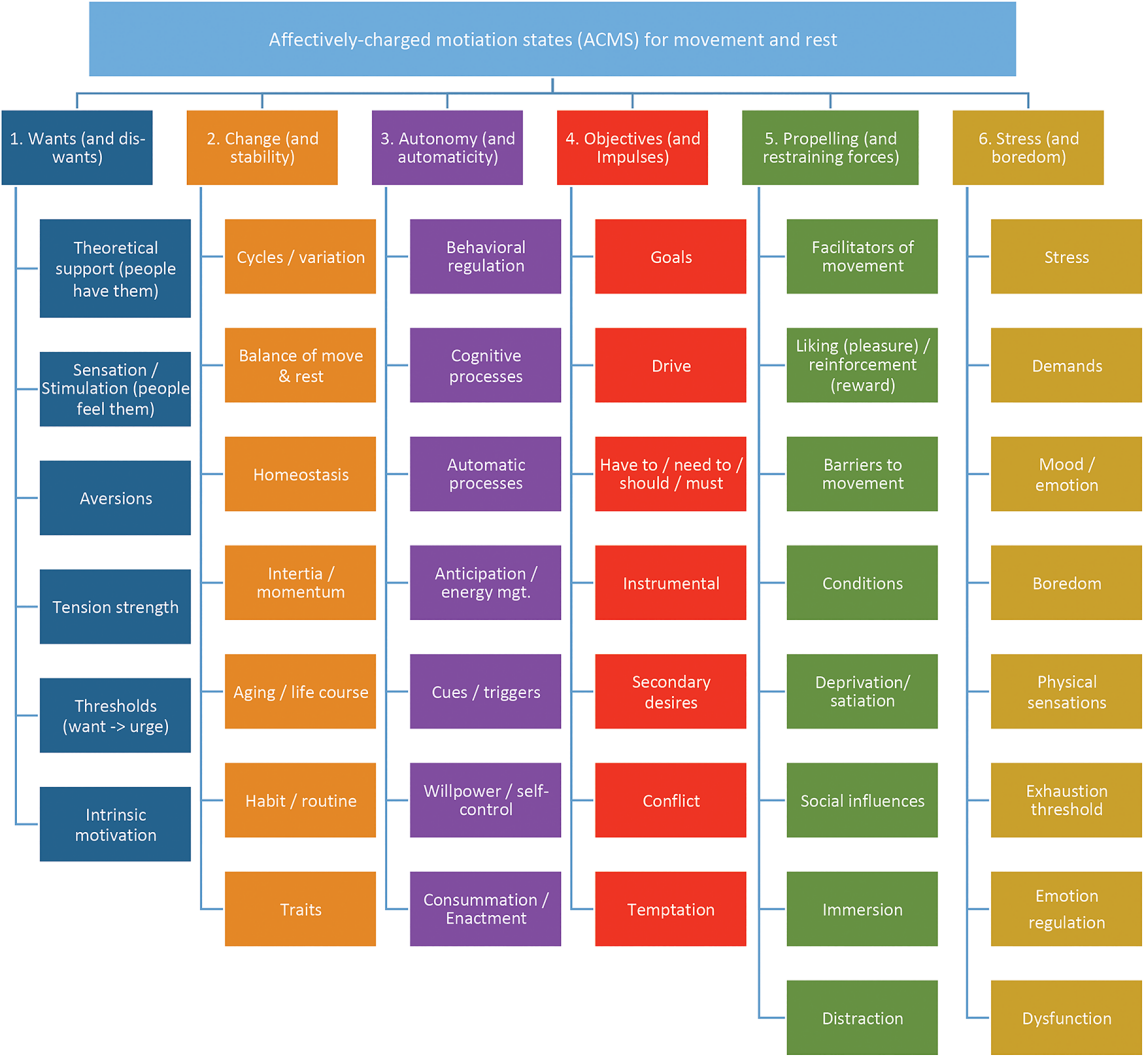
Final model of the 43 higher order themes (HOTs) categorized into six super higher order themes (SHOTs).

We then decided to present the themes as dualities based on the contrasting ideas of: (1) “propelling vs. restraining” forces, (2) “automaticity vs. deliberation” in the Affective-Reflective Theory of Physical Inactivity (14), and (3) “reflective vs. appetitive” desires (12, 18). However, to be consistent with the WANT Model (17, 20, 24), we decided to present these dualities as additive (“and”) and not necessarily as a conflicting binary (“vs.”). This was also done to emphasize the potential for an adaptive and flexible behavioral repertoire (47), as with the WANT Model (i.e., which includes move and rest, and not move vs. rest), where combinations of desires can lead to more diverse behavioral outcomes (47). The final SHOTs were: (1) Want—and do not want (diswants), (2) Change and stability, (3) Autonomy and automaticity, (4) Objectives and impulses, (5) Restraining and propelling forces, and (6) Stress and boredom. Each SHOT is explained in detail below. See Figure 2.

#### SHOT 1—Wants and diswants (aversions)

All of the higher order themes in this SHOT related to affectively-charged motivation states and their characteristics. Thus, the SHOT was called “Wants and diswants”. This SHOT encompassed six higher order themes: (1) sensations/stimulation (i.e., people subjectively feel wants to move and rest), (2) theoretical support for ACMS and the WANT model, (3) tension strength of motivation states, (4) aversions/dread for movement and rest, (5) intrinsic motivation, and (6) thresholds differentiating ACMS. Supplementary Table S1 contains the HOTs, exemplar LOTs, and quotes for the “Wants and diswants” super higher order theme.

#### SHOT 2—Change and stability

The higher order themes in this SHOT all related to factors changing or remaining stable over time, which invokes concepts of biorhythms and regulated change, and perhaps similar to homeostasis and allostasis (48). This SHOT had seven higher order themes: (1) cycles and variation, (2) homeostasis, (3) inertia and momentum, (4) balance of movement and rest, (5) habits and routine, (6) traits and (7) aging and the life course. Supplementary Table S2 contains the HOTs, exemplar LOTs, and quotes for the “Change and stability” super higher order theme.

#### SHOT 3—Autonomy and automaticity

With close inspection of the HOTs, it was determined to call this SHOT “Autonomy and automaticity”, in respect to processes of control and higher-order cognitive processes, such as decision-making. Both HOTs and LOTs in this theme appear to point to many user-generated decisions—or at the very least—the inability to make decisions (49). In other words, some processes have a locus of control generated consciously by the self, while others are generated more unconsciously. Considering the framework of Self-Determination Theory (44, 50), the notion of autonomy is a feeling that one has choice and is willingly endorsing one's own behavior (49). Autonomy here makes sense as individuals might think about actions (e.g., cognitive processes), regulate behavior (e.g., energy management), and must overcome urges in order to produce a desired behavior (e.g., temptation vs. will power). When looking through this lens, autonomy appears as the concept that encompasses all of these lower order themes. In regard to those decisions that appear to be not consciously generated, the idea of automaticity applies from Affective-Reflective Theory (14). Recent work suggests that automaticity and autonomy interact to produce stronger physical activity behaviors (51). This SHOT had seven higher order themes: (1) automatic processes, (2) cognitive processes, (3) behavioral regulation, (4) anticipation/energy management, (5) cues/triggers/feedback, (6) willpower/self-control, and (7) consummation/behavioral enactment. Supplementary Table S3 contains the HOTs, exemplar LOTs, and quotes for the “Autonomy and automaticity” super higher order theme.

#### SHOT 4—Objectives and impulses

This SHOT was named “objectives and impulses” to reflect that some desires are rational while others are appetitive, which is in line with the theory of desires from Davis (12, 18). Examining the lower and higher order themes of this SHOT, and the quotes associated with them, it was apparent that the respondents were trying to reconcile commitments against impulses to accomplish competing objectives. This would entail setting and working toward goals, in concert or in conflict with thinking about what one has to do, needs to do, should do, or must do based on the desired end result. Taken together, this reads as an objective-oriented mindset contrasting against an impulsive mindset, where one is actively working towards a desired outcome. This SHOT had seven higher order themes: (1) goals, (2) drive, (3) have to/need to/should/must, (4) instrumental demands, (5) conflict, (6) secondary desires, and (7) temptation. Supplementary Table S4 contains the HOTs, exemplar LOTs, and quotes for the “Objectives and impulses” super higher order theme.

#### SHOT 5—Propelling and restraining forces

Respondents indicated that there were several factors that modified or moderated their experience of motivation states to affect activity and sedentary behaviors. Thus, it was decided to name this “Propelling and restraining forces”, in alignment with Affective-Reflective Theory (ART) of physical inactivity and exercise (14). This SHOT had eight higher order themes: (1) facilitators of movement, (2) deprivation / satiation, (3) barriers for movement, (4) social influences, (5) conditions, (6) liking (pleasure) / reinforcement (reward), (7) immersion, and (8) distraction. Supplementary Table S5 contains the HOTs, exemplar LOTs, and quotes for the “Propelling and restraining forces” super higher order theme.

#### SHOT 6—Stress and boredom

Respondents frequently noted that desires to move and rest were instigated by states of over- and under-stimulation—strain and monotony. This makes sense, as psychological stress states can have a strong effect on physically activity and sedentarism (52), both inhibiting and activating behavior, perhaps by affecting psychosomatic sensations (53). This SHOT encompassed eight higher order themes: (1) impinging life demands, (2) physical sensations, such as energy and fatigue, (3) stress, (4) exhaustion threshold, (5) monotony and boredom, (6) emotional regulation, (7) mood and emotion, and (8) dysfunction and dysregulation. Supplementary Table S6 contains the HOTs, exemplar LOTs, and quotes for the “Stress and boredom” super higher order theme.

## Discussion

This is the first mixed-methods (qualitative and quantitative) study to provide evidence that individuals experience appetitive and reflective wants (or desires) to move and rest; that these states change rapidly, and are highly influenced by a number of ever-changing factors, such as the daily experience of stress (52). Interviews with 17 college honors students revealed that they subjectively felt affectively-charged motivation states (ACMS) to move and rest both in recent weeks and in the present moment. They also provided evidence for other postulates of the WANT model, such as not wanting to move or rest at all, or rather, *actively avoiding* certain behaviors. In a few cases, respondents provided contrasting perspectives that contradicted expectations. For instance, some respondents expressed some doubt that they had any desire to move, or that there were any differences between various motivation states, such as the desire to move vs. an urge to move. Importantly, some evidence, though not extensive, supported the idea that motivation states to move and rest spur actual activity behaviors in a time frame proximal to the experience of the subjective desire. To understand how motivation states might impact behavior, we conducted an inductive content analysis of the interviews. Forty-three higher order themes were found, which we separated into six super-higher order themes, such as “Objectives and impulses”, “Propelling and restraining forces” and “Stress and boredom”. The study also provided further validation of the CRAVE scale, which was recently developed to measure motivation states (24, 26). As measured with this instrument, there were changes in ACMS from pre- to post-interview with moderate effect sizes (0.76 and 0.50, for move and rest, respectively). Overall, there was an abundance of support, but also some minor conflicting evidence, for the concept of affectively-charged motivation states for physical activity and sedentarism and their influence on subsequent behaviors.

### Evidence of affectively-charged motivation states—quantitative and qualitative deductive analyses

The major priority of this study was to determine if a group of respondents would qualitatively support or negate the idea of feeling motivated, in the present moment, to move, be active and exercise. This has come into focus given a preponderance of opinion, and some empirical data, that humans prefer to be sedentary, or may not have any experience of desiring or wanting movement (35, 36). While there is a strong rationale that humans do want to move (17) and initial quantitative data exists to support it (24), we were interested in opinions from interviewees and their expressions of desire (or lack thereof) in their own words. As it happened, participants largely corroborated the concept of motivation states, but they also presented unique perspectives. Participants stated that they did subjectively experience desires to move and rest, including at the time of the interview, which sometimes differed from desires experienced over the past week. Moreover, these states were volatile, rapidly dissipating or succumbing to other desires. They also described a total absence of desire, often during flow states, or even aversion to movement and rest; in other words, actively avoiding these behaviors. Desires were sometimes described as being consummatory (e.g., as in feeling an urge to exercise that instigates actions to go work out at the gym), which in turn often resulted in satiation—a fulfillment of desire leading to a drop in the motivation state and subsequent cessation of activity. Of note, strong urges and cravings for movement, typically from conditions of deprivation (e.g., sudden, abnormal, and/or prolonged decreases in exercise) were associated with physical and mental manifestations, such as leg stiffening, fidgeting, and feelings of being antsy, jittery, and restless. Urges for rest and sleep featured prominently as well, and some respondents even expressed having extreme cravings for rest.

While qualitative data addressed the experience of motivation states, quantitative data mainly demonstrated variance in those states. As hypothesized, motivation states, as assessed with the CRAVE scale, changed significantly over the course of the focus groups. We observed that the desire to move increased pre- to post-interview, and the desire to rest decreased, which agrees with data previously collected from a study that saw similar trends over three time points throughout an educational seminar (24). Some participants described the experience of motivation states as something novel and unimaginable beforehand, but now that it was in their conscious awareness, it had some concreteness and veracity. The notion that we may or not be aware of our desires and impulses has been discussed extensively (30, 31), and the idea of arousing awareness for movement impulses was famously demonstrated by Benjamin Libet (54) in his studies on free will. Having satisfactorily analyzed the interviews for deductive evidence of motivation states, we turned to analyze the data from an inductive perspective, creating hierarchical themes to understand how motivation states operate within a larger regulatory scheme. The first super higher-order theme, “wants and diswants (aversions)” was mostly constituted from the deductive information generated above.

### Qualitative inductive analysis

Do motivation states matter in the control of behavior, and if so, how? Desires and urges for movement and rest are ostensibly antecedents to and consequences of behavior, but how they operate within behavioral systems is unknown. Super higher-order themes two through five commonly related to ideas of behavior regulation, comprising the categories of: SHOT 2) Change and stability, SHOT 3) Autonomy and automaticity, SHOT 4) Objectives and impulses, and SHOT 5) Restraining and propelling forces. In regard to the SHOT on change and stability, participants widely reported diurnal, weekly, and seasonal variation in desires for movement and rest. A plethora of data exists in the area of circadian rhythms (26), supporting the notion that pertinent hormones (e.g., cortisol) (55), neural peptides (e.g., hypocretin-1 / orexin A) (56), psychological factors (e.g., perceptions of energy and fatigue), and other attributes vary cyclically over the course of a day, month, year, or longer (23). Another theme emerging from this SHOT was that of behavioral and motivational momentum and inertia (e.g., being “in a rut” or “stuck”) (15), perhaps similar to the ideas of affective inertia or *stickiness* (57), which are associated with symptoms of depression and/or attention disorders (58). This is intriguing as one might speculate that motivational inertia serves as another indicator of psychological dysfunction. On the other hand, the feeling of inertia may be a key difference between types of ACMS, with cravings and urges having more motivational pull against inertia. Clearly though, sometimes inertia is less pernicious and simply due to forces of habit and environmental demands. Participants indicated that habit drove their behavior without awareness of a desire for movement, attesting to the power of habits (59, 60). Another force (perhaps equally strong to habit) indicated by respondents was provided by movement's instrumental or utilitarian value. In short, despite technological advances, people still have some tasks that can only be accomplished through bodily movement, and motivation states match those situational demands through a process Brehm and Self (29) call *motivational arousal*.

These concepts segue easily into SHOT 3 (Autonomy and automaticity), and of these two contrasting perspectives, perhaps automatic processes of regulation were most frequently described by participants; they did represent the greatest number of lower-order themes in this SHOT. Central to automatic processes are the related ideas of randomness and spontaneity, which recently have been highlighted in motivation research but are rarely accounted for in analyses (61); “Motivation arrives as opposed to being planned” (61), suggesting a non-linear path of motivation. Participants were clear that a variety of external variables, such as cues, were antecedents of movement and rest, perhaps accounting for some of this variability (15). Finally, the theme of automaticity is also consistent with terminology used in the social psychology work on conscious vs. nonconscious processing and decision-making. Hallmark research from Bargh (62) resulted in the adoption of *automaticity* vs. *control* to refer to non-conscious and conscious processes. In the current case, autonomy and control are parallel in that they refer to conscious, volitional processes.

In line with the Affective—Reflective Theory of Physical Inactivity and Exercise (14), participants also described, though less frequently, deliberative processes, such as decision making, planning, energy management, and prioritization of rest and exercise. Motivation states are key mediators in adaptive planning (47). As seen in SHOT 4, participants described desire changing in the context of conflict, which existed between competing desires as well as between desires and goals. Participants expressed these as contrasts between “want to”, “have to”, “should”, “need to”, and their converses (“don't want to”, “shouldn't”, etc.), many or most of which interacted with goals, intentions, and other cognitive factors to spur change (17, 63–65). Saunders and colleagues (66) found that, on average, 60% of participants' desires conflicted with at least one goal. The interplay between these forces was often influenced by willpower (resistance), self-control, or harmonization of desires—to result in behavioral enactment or avoidance. Greater resistance or willpower applied immediately in the moment of temptation results in less enactment of unproductive desires (66, 67). While a lack of willpower might be the key factor in some situations, in others it might more simply be a lack of opportunity (e.g., situational constraints) to move or be sedentary in the moment of experiencing desire. Sometimes respondents indicated that desires were managed, manipulated, or ignored, but frequently urges and cravings were strong enough to hijack attention and thoughts, consuming physical and mental resources to the point of not being able to overcome them—ostensibly resulting in rapid behavior (63, 68). On the other hand, as laid out in SHOT 5, there were a variety of barriers blocking consummation of the desire to move, such as injury, exhaustion, and responsibilities. There were also a variety of conditions (e.g., having free time for leisure, being in proximity to a gym or nature) and social factors facilitating desires—leading to opportunities to act on the impulse (11, 49, 69). Taken together, motivation states appear to play a prominent role in behavioral processes. More specifically, they seem to relate clearly to the concept of self-regulation, which is defined as “any effort to actively control behavior by inhibiting dominant and automatic behaviors, urges, emotions, or desires, and replacing those with goal-directed responses” (70, 71).

### Stress and boredom

Psychological stress, both subjective and objective, emerged as a major theme, and participants frequently cited facets of stress as abating and/or instigating motivation states to move (e.g., “Stress makes me want to move.”) and rest (e.g., “My desire to rest is normally about stress.”). Stressful emotions (e.g., “freaking out”, being overwhelmed), life stressors (e.g., transition to college, COVID-19, family death), demands (e.g., schoolwork, sports training), daily hassles, and work/rest imbalance were all regarded as influential in either activating or inhibiting motivation and related behavior. Several participants stated that they utilized exercise as a method to cope and regulate emotions, which may explain why some people move more in the face of stress. All of these observations fall in line with a classic systematic review that found that psychological stress was associated with inhibited physically active behaviors in 86% of higher-quality studies, but 18% of prospective studies found that it was associated with activated movement as well (52). Investigations including sophisticated analyses have demonstrated that the effects of negative affect on physical activity are stronger than the opposite direction (72, 73). Stults-Kolehmainen, Blacutt & Filgueiras (74) found that individuals reporting very high levels of stress reported either no exercise at all, or alternatively, very high levels of exercise (e.g., working out 6 days a week). Despite facing extraordinary stressors, some athletes are able to self-regulate to maintain effortful behaviors by focusing on goals, the so-called “self-regulatory efficacy” (75), resulting in a null effect of stress.

Back to the current data, excitement and eustress typically were related to an increased drive to move (21), but so was a lack of stress and under-stimulation—feelings of boredom and monotony. Interestingly, stress also resulted in feelings of numbness or being frozen, in other words, not wanting to move or rest at all, which is in accordance with postulates of the WANT model (17). One unique observation was that not being able to satisfy or consummate an urge or craving to move or rest sometimes resulted in the experience of stress, frustration, and agitation, indicating possible bidirectional effects (e.g., “I couldn't satisfy the urge to move and get stuff done, and that was very stressful”). Overall, it appears that stress and emotion interact with motivation states (e.g., desire, urge, craving) to move and rest in a highly complicated manner to influence behavior (76). Unfortunately, at the current time there is a lack of a clear model to explain stress and motivation interactions—whether motivation mediates and/or moderates the effects of stress on physical activity.

Aside from psychological stress, other mental health and psychological considerations had sway over motivation states. For instance, psychosomatic sensations, such as tiredness, pain, and soreness all had a clear impact on desires to move and rest, with aversive sensations typically extinguishing the desire to move and propelling desires to be sedentary. Both good and poor moods were commonly cited as influencing desires to move, be productive, and rest. Although unprompted, some respondents openly commented that they had various mental health conditions, such as anxiety, ADHD, bipolar disorder, and body image problems. These respondents spoke about episodes of impaired activity—being “in a rut”, feeling “stuck” or, conversely, being hyperactive and feeling manic (77). However, no participant discussed depression and trauma. Those with PTSD, for instance, sometimes complain of being “frozen” and unable to move and be productive (78), while those with panic attack and agoraphobia suffer from “fear responses to acute threat with the urge for active avoidance/escape” (79). Stults-Kolehmainen and colleagues have discussed aspects of motivation states as they appear in psychological disorders, including: anorexia nervosa, muscle dysmorphia, akathisia, restless legs syndrome, and others (20). Until recently, these sensations appeared to be obscure and idiopathic symptoms, but recently NIMH has classified these in the *sensorimotor domain* under the construct “motor actions” (sub-construct: “sensorimotor dynamics”) (80), which seems to validate the notion that ACMS might have a place in mental health and pathology. Unfortunately, this study included a sample that was too small to explore any of these ideas, and we did not include any physical or mental health measures in this study.

### Limitations

The results of the current investigation must be interpreted with some caution due to several limiting factors. First, the number of participants was small and homogeneous; the group was composed mostly of young, female, undergraduate honors students. Previous studies have found no differences between genders for motivation states (24), so lack of variability in gender may not be an issue, but we have observed differences by age (24). Older individuals have a much wider range of life experiences and are subject to the effects of both primary and secondary physical aging (81). Consequently, it seems likely that older adults will experience motivation states for movement differently and will probably have a greater desire for rest. Comments from our respondents could also reflect a specific motivational climate, culture, and education around movement and rest that might be tied to this population of high achieving college students (49, 69, 82). Indeed, our previous work also demonstrates that adults of different age groups have different motives for exercise (10). Motivation states also vary by exercise stage-of-change, a proxy for physical activity behavior (24). The fitness and physical activity levels of the sample were not measured, but based on their extensive comments, it is certain that this group was a healthy, active, and high functioning sample. This is important, as for some people, there are likely trait manifestations of wanting to move and rest.

Regardless of these influential factors, the interviews generated over 400 lower-ordered themes, indicating that even though the participant number was low, the interviews were very productive. Also, despite the low n, we observed changes in CRAVE scores across the focus groups, indicating increased desires to move. There was no control group, therefore, we don't know if changes in CRAVE (“right now”) were due to: (1) increased awareness of normally unconscious desires, resulting from talking about physical activity and rest behaviors (54), (2) the effects of behavioral priming, which Bargh demonstrated clearly impacts physical activity (62), because participants felt “cooped up” during the interview, (3) anticipation of leaving the venue for their next daily task, (4) demands effects, (5) reactivity to the CRAVE scale, or (6) some other unknown factor. The first point might be discounted as we did not observe concomitant increases in the desire to rest (“right now”), even though it was also widely discussed. On the contrary, it was diminished. Furthermore, we found similar results in Study 4 from Stults-Kolehmainen et al. (24), which observed a lecture period when the topics of physical activity and rest were not specifically discussed. Future studies will need to untangle these effects with better experimentation.

### Future research

Future research could attempt to make the necessary methodological advances noted above, or it could go in alternative directions to address other issues, which are roughly divided into four research questions.

#### Are people naturally lazy?

While the current study provided both quantitative and qualitative evidence that desires to move and rest are subjectively felt in conscious awareness, and most aspects of the WANT model were supported, we were not able to adequately address the idea of which desire (physical activity or sedentarism) is predominant in this group of respondents, nor in the larger scope of human behavior. However, our quantitative data found that the desire to move was greater than the desire to rest at every time point. This is consistent with our previous investigations, where desire to move was consistently rated higher than desire to rest in quantitative analysis (24). It is also congruent with the idea that, “The human body is built for physical activity, not rest” (83), implying that humans have both a natural need and inherent drive to move [discussed extensively by Stults-Kolehmainen et al. (17, 21)]. However, we did not specifically ask our interviewees questions to directly compare desires, such as, “Which desire do you feel more often?” or “Which desire is stronger for you typically and right now?” Various researchers have suggested that humans are naturally inclined to rest and thus conserve energy; therefore, they likely have greater desire to be sedentary, are typically lazy, and only move when necessary (12, 29, 35–37, 84). The ideas of laziness and productivity did feature among respondents in these focus groups, with laziness generally being viewed as the opposite of productivity, and desires to rest and move associated with those tendencies (e.g., “I feel urged to move and get stuff done.”). Future research should address whether the feeling of laziness is simply: 1) a lack of a desire to move (regardless of the desire to rest), 2) a combination of low desire to move and high desire to rest, or 3) a low desire to move and a high desire to rest but felt in the shroud of “should” move.

#### How do “shoulds” and “want to's” interact?

Following from above, a person may feel and/or think that they “should” be moving, and they “should” be productive, but they do not have the subjective and appetitive feeling of wanting to move and be productive. Future studies should address the ideas of “should” and “have to” in relation to reflective and appetitive “want to”—developing better instruments and theories to connect these related constructs. More practically, future studies might investigate how to create exercise routines that are more enjoyable, less compelled by “shoulds”, or help people to move more mindfully—paying attention to desires and/or embracing desires to move and rest in balance, as with mindful walking or martial arts (85, 86). These might be conducted as part of just-in-time adaptive interventions (JITAI) (87), which attempt to gauge and take advantage of motivation states (i.e., “CRAVE moments”), but at this time no studies have sufficiently incorporated this idea (88)—as none have used a valid measure, such as the single-item CRAVE scale (24, 27).

#### How can the WANT model be improved?

Future research should also focus on updating and revising the WANT model (17), which was created because of apparent theoretical deficits and the inability of existing theories to adequately explain the motivation states phenomena we have observed, but still falls sort of its intended goal. In short, the WANT model is a heuristic to understand how desires might vary in strength, approach, and how they interact with each other. This effort is concordant with the NIMH Research Domain Initiative Criteria, which seeks to understand elements of psychological phenomena, such as urges and motor sensations (80). The current data provide evidence that facets of the WANT model (e.g., that desires may be oriented to approach or avoidance, are impacted by previous behaviors, etc.) are valid. However, the model as a whole lacks extensive quantitative validation, mainly because there are no measurement tools available to assess aversions (i.e., diswants) to move and rest. Until this measure exists, adequate validation of the dual-axis structure of desires for movement and rest (and thus four quadrants) is not feasible. The WANT model could be further explicated by considering how ACMS to move and rest fluctuate in tandem with other internal and external states (e.g., stress, satiety, deprivation, hunger, and fatigue), emotion, and other influences that vary by the situation and context. For example, a revised WANT model might incorporate exogenous stimuli known to influence desires to move, such as music, light, and other environmental factors identified by participants in these focus groups. Insights generated from qualitative data, as in the current study, is pertinent for the advancement of model, theory, and intervention development in the areas of physical activity and sedentary behavior, as has been delineated by Bonell (40).

#### What other theories offer insight into motivation states for movement and sedentarism?

The WANT model is not a predictive model; therefore, frameworks predictive of physical activity and sedentarism, such as dual-process theories (14, 15), or models of affective response (16) may be better suited to explain these phenomena. Alternatively, new research frameworks may be needed. Dissimilar from theories mentioned above, the Elaborated Process Model of self-regulation (33) focuses on the idea of depletion and how it moderates motivation. It asserts that people switch from move to rest (and from “have-to” to “want-to”) systems and back again as they become depleted in each system. Importantly, depletion is associated with fatigue, boredom, and negative emotions, which propels the individual to avoid exploitation types of tasks (i.e., work) and approach exploration types of tasks (e.g., watching video clips or television) or vice versa. While the switch is ostensibly prompted by fatigue, it may also be triggered by a (perhaps unconscious) cost/benefit analysis, stoking desires for rewarding stimuli and causing changes in attention, salience, and emotion. From an evolutionary aspect, such fatigue-induced switching is highly utilitarian and adaptive as it: (A) prevents excessive focus on any one single desire, for instance, in the dysfunctional cases of *punding* (89), and (B) it also protects the human organism from overexertion and collapse (90, 91). However, the theory also postulates that to promote the continuation of valued behaviors, fatigue can be better tolerated and made less aversive with the provision of extra reward, distractions, affirming values, prayer, or other strategies (33), all of which may be relevant in the promotion of physically active behaviors. Other models of depletion and satiation, such as the exercise satiation model (92), should be studied for ideas to expand and/or modify existing frameworks—together with Self-determination Theory (44, 50) and the Theory of Effort Minimization in Physical Activity (36).

Finally, future research should investigate whether people act on their desires to move and rest when these are experienced in the moment of tension. This may be studied in a naturalistic setting (93) or in a laboratory environment where such desires are instigated.

## Conclusion

Both quantitative and qualitative data support the notion that humans experience subjective feelings of wanting or desiring to move their bodies, be physically active and/or exercise, which we call affectively-charged motivation states (ACMS). Sometimes, these actionable feelings were described as strong, engrossing or even irresistible, as in an urge or craving to get up and walk around or engage in a workout or training session. Respondents clearly indicated having experiences of desire or craving to rest, sleep, and engage in sedentary behaviors, and frequently these collided with ambitions to move or be productive. How interviewees described their subjective states largely fell in line with postulates of the WANT model (17), which describes how desires, wants, urges, and cravings to move, be active and rest operate loosely and asynchronously. Motivation states to move and be sedentary varied by numerous factors, which we divided into six super higher-order themes. Perhaps the most prominent of these was the theme centered on stress. Indeed, the experience of stress frequently stymied desires to move and be active, though sometimes it had the opposite effect. Stress also stoked desires to rest and be sedentary, though again, sometimes it also diminished those. Quantitative data revealed that, across focus groups, desires to move increased, and desires to rest trended to decrease, which participants corroborated when specifically asked about perceived changes in motivation states. The overarching picture that emerged from this investigation is that motivation states (e.g., desires, wants, urges, and cravings) likely play a prominent role in behavioral processes, interacting with other factors (e.g., stress, habit) to drive movement and sedentary activities. Such information may lead to better theories and, down the road, adaptive interventions to promote physical activity.

## Data Availability

The raw data supporting the conclusions of this article will be made available by the authors, without undue reservation.
