# Supplementary material for: Qualitative and quantitative evidence of motivation states for physical activity, exercise and being sedentary from university student focus groups
Source: Front Sports Act Living. 2023 Mar 21;5:1033619. doi: 10.3389/fspor.2023.1033619 (PMC10071436; doi:10.3389/fspor.2023.1033619)
Supplement: Supplementary file 1 [file Table1.pdf]

Supplementary Table 1. Super Higher Order Theme 1: Want – and do not want

#	Higher order theme (H.O.T.)	Explanation	L.O.T.s attributed to this H.O.T. (count) *	Exemplar L. O. T.s **	Exemplar Quotes ***
1	Sensations / stimulation	People subjectively feel wants to move and rest	62	Feeling restless;<br>Feeling jittery;<br>Feeling antsy; "cooped up"<br>Feeling stiff from sitting a long time;<br>Nervous tension	"I've been pretty **restless** lately, so I've been running around in my dorm, organizing things." (9/8, A)<br><br>"Last night … I was just awake and had this **jittery-ness** - almost where it was hard for me to fall asleep. I didn't want to rest." (9/17, A)<br><br>"…after not doing anything all morning, and now I'm actually feeling a little bit **antsy**…" (9/17, A)<br><br>"When we are sitting doing homework for a really long time, I wanna be more physically active or just move around so that my legs or [all of my body] isn't **stiffened up.**" (8/32, D)
2	Theoretical support	Support for the concept of ACMS and the WANT model	37	Theory (for WANT model);<br>People actually have desires to move;<br>Cravings for rest exist;<br>Wants to take a nap;<br>Rest more than move;<br>People have aversions for movement;<br>Don't want to rest;<br>Wants to move vary in intensity;<br>Don't want to do anything at all, including move or rest;<br>Wants to move influence behavior;<br>Lack of desire to rest --> affects sleep;<br>Feel an enhanced urge to move or rest	See Tables 3 and 4 above.
3	Tension strength	ACMS range from very weak to very strong in magnitude	17	Weak vs strong tension;<br>Desire- not strong enough to make movement happen	See Table 4 above.
4	Aversions / dread	People have active motivation to avoid movement and rest	11	Aversion to move;<br>Wanted to not move;<br>Can't rest	See Table 3 above.
5	Intrinsic motivation	Similar to concept of intrinsic motivation in Self-Determination Theory.	7	Wants/desires to move associated with anticipations of pleasure, fun and enjoyment;<br>Desire to scavenge, be curious, explore, move about;<br>want - movement for its own sake, not to accomplish something else	"Sometimes, running in the rain is fun." (9/15, A)<br><br>"What makes me want to move is just the joy I get from playing sports. I enjoy exercising, weight training, conditioning, running, stuff like that. I would definitely feel more motivated by playing sports." (9/17, A)<br><br>"Soccer playing and training is the most enjoyable part of my day." (9/17, A)<br><br>"I urge myself to move because I know that I need to … get myself to walk around and get some fresh air or just experience all of the things around me." (9/13, A)
6	Thresholds differentiating ACMS	Desires and wants are weaker than urges and cravings	5	Urge - have to cross a threshold of exhaustion;<br>Urge / crave has greater intensity and similar to a "need to";	See Table 4 above.

		Very similar constructs
	TOTAL = 139	

* These are the number of LOTs originally attributed to this HOT theme by analysts. During stages of re-review, some LOTs were reassigned to different HOTs for coherence, which may slightly change the quantity of LOTs in the following column.

** Many LOTs can (and may be) cross loaded onto other HOTs.

*** Many quotes can be cross loaded onto other HOTs, but efforts were made to place unique quotes only into 1 (or two) HOTs.
