# Supplementary material for: Qualitative and quantitative evidence of motivation states for physical activity, exercise and being sedentary from university student focus groups
Source: Front Sports Act Living. 2023 Mar 21;5:1033619. doi: 10.3389/fspor.2023.1033619 (PMC10071436; doi:10.3389/fspor.2023.1033619)
Supplement: Supplementary file 2 [file Table2.pdf]

Supplementary Table 2. Super Higher Order Theme 2: Change and stability

#	Higher order theme (H.O.T.)	Explanation	L.O.T.s attributed to this H.O.T. (count) *	Exemplar L. O. T.s **	Exemplar Quotes ***
1	Cycles / variation	Psychological and biological rhythms and regulated change	35	Seasonal variation / holidays / transition to college;<br>Weekly variation (e.g., it's a Monday);<br>Work schedule;<br>Stability or consistency of schedules (e.g., sleep);<br>Just woke up;<br>Time of day (e.g., AM / PM, sunrise / sunset)	"Later in the day is just when I really want to rest." (9/17, B)<br><br>" … I had this really big burst of energy over the weekend, and now I am falling back down again - so I am trying to get back up so I can be healthy and work out." (9/21, A)<br><br>"I remember all of last summer, every morning, I craved to workout, and I craved to practice even though I couldn't go. In my own time in my backyard I would work out and mimic a practice by myself because I craved it." (8/31, B)<br><br>"In the mornings I really wannabe active" (8/31, D).
2	Homeostasis	Processes of returning to a set point	25	Can sleep better if has moved;<br>Got a sufficient (or an excess) of sleep last night;<br>Need to recover (so can be more active);<br>Overexertion -> need rest;<br>Under exertion -> need to move more;<br>Crave - return to homeostasis;<br>Desire - move to return to homeostasis	"If I worked out previously, I probably just want to rest, and if I have a big exam, I want to do that and later devote my energy to working out." (9/10, B)<br><br>"The workload is not too heavy -- so I am feeling like I wish we were doing a bit more." (9/17, A)
3	Inertia / momentum	Movement begets more movement and sedentarism begets more sedentarism	15	Have just been moving;<br>Resting and being lazy;<br>Wanting to move, but lacking energy<br>Feeling "Stuck"; stuck in a rut, trapped, frozen;<br>Inertia and momentum;<br>Sometimes can't move;<br>Inertia;<br>Wanted to keep moving after school activity;<br>Want to move on a busy day;<br>Momentum;<br>Move - brain won't "shut-off";<br>Want to continue moving;<br>Want to continue moving	"I would say yes [I want to move right now] and that is partially because I walked here, and I have already started to move." (9/10, A)<br><br>"I have ADHD … I will find myself stuck in a rut, and I'm just, watching TV, reading a book, something like that, which is fine and all, but I'll be doing it for hours...[but] I want to work out, or I want to take my dog on a walk. There's nothing in the world that is making me not move, it's just my brain just gets like stuck - almost frozen. Usually, it's not something like outside that's, stopping me from doing it. It's myself. Like when an object, in motion...tends to stay in motion. If I stop, I just know I'm not gonna be doing anything for the rest of the day, unfortunately. It kinda feels like sleep paralysis sometimes where you know it's happening, you want to make it stop, or you want to move, but you just cannot. It's a little depressing [laughs], you know? [I'm thinking], "I wish I could". (9/10, A)<br><br>"Getting up and moving is harder [when not already moving], but when I have a busy day, I want AND desire to move." (9/10, A)
4	Balance of movement and rest	Periods of rest are necessarily followed by periods of movement and vice versa.	11	Desire to move - when not moving;<br>Laziness;<br>Being sedentary;<br>Crave movement with absence of movement;<br>Want rest after movement;<br>Mental break needed;<br>Move/rest balance;<br>Movement can be a source of rest	"There are days when I wanna move more, because I've been resting, and there are other days when I wanna rest more because I've been moving." (8/31, A)<br><br>"When you've been sitting in class for a while, you just wanna move around." (8/31, A)<br><br>"For me, resting is sometimes going out on a walk and just enjoying the outside. I don't need to rest by sitting down because for me being active also gives me some rest because it lets my mind go free." (8/31, B)<br><br>"I feel sometimes for me mental rest comes with physically being active. I can just exert myself without having to think about school." (8/31, D)

					"Craving is more when I am doing something [highly] repetitive because I am bored of the same activity, so I want to do something else, if that's resting, being on my phone, or just laying down or watching TV. While want and desire is when I am doing something in the moment - let's say I am working out, and I think 'Oh, I want to stop'". (8/31, B)<br><br>"I need the rest to be able to be more active." (8/31, B)
5	Habit / routine	Regularity of health behaviors	11	Habit / routine<br>Sleep schedule / routine / consistently of schedule<br>Weekly schedule<br>Self-care	"I usually like to go to the gym and run a lot." (9/15, A)<br><br>"I have wanted to rest a lot, and that is because I just don't have a good sleep schedule." (9/10, A)
6	Traits	Enduring characteristics of a person	2	Desire to rest as a trait, not a state<br>Desire to move as a trait, not a state	"I'm also a morning person so I wake up at 6am." (9/21, A)<br><br>"I [wanna] be that person who enjoys running in negative-ten-degree weather" you know?" (8/31, D)<br><br>"I'm the kind of person who likes to sleep." (9/13, A)
7	Aging / life course	Effects of again on motivation states	1	Age / being a teenager or young adult	Interviewer: "… what …. made you want to rest [last week]?<br>Participant A: "… just [being] like a normal… person… not person but [a] normal teenager." (9/13, A)
			TOTAL = 100		

* These are the number of LOTs originally attributed to this HOT theme by analysts. During stages of re-review, some LOTs were reassigned to different HOTs for coherence, which may slightly change the quantity of LOTs in the following column.

** Many LOTs can (and may be) cross loaded onto other HOTs.

*** Many quotes can be cross loaded onto other HOTs, but efforts were made to place unique quotes only into 1 (or two) HOTs.
