# Supplementary material for: Qualitative and quantitative evidence of motivation states for physical activity, exercise and being sedentary from university student focus groups
Source: Front Sports Act Living. 2023 Mar 21;5:1033619. doi: 10.3389/fspor.2023.1033619 (PMC10071436; doi:10.3389/fspor.2023.1033619)
Supplement: Supplementary file 3 [file Table3.pdf]

Supplementary Table 3. Super Higher Order Theme 3: Autonomy and automaticity

#	Higher order theme (H.O.T.)	Explanation	L.O.T.s attributed to this H.O.T. (count) *	Exemplar L. O. T.s **	Exemplar Quotes ***
1	Automatic processes	Effects of automaticity, randomness and spontaneity	15	Random bursts of energy;<br>Randomness depends on the day;<br>Spontaneity;<br>Randomness: Some days need to move, some need to rest;<br>Start taking action when feel an urge;<br>Urge & crave: These are when there is a certain course of action determined;<br>Want happen automatically at certain times	"I get like random bursts of like energy, and it makes me really motivated, and it like makes me want to get more done, and it makes me wanna go on runs, go on walks, get more homework done, or get the next week's homework done." (9/13, A)<br><br>"… You've been doing something, like studying, for a while, and you just get a random burst of energy, and then you run around your dorm." (9/8, A)<br>"… Sometimes I get the urge to [move]. It just depends on the day." (9/17, C)<br>"We all have instances when we want to get up and do things, but we all have instances where we just want to sit around and watch TV for a day or something." (9/8, A)
2	Cognitive processes	Thoughts and higher order thinking.	14	Highly focused on desire / cognitive hijacking;<br>Cognition - think about it more when you can't do it;<br>Desire - triggered by memories of former athletic self;<br>Urge - thinking about it more and obsessive thoughts;<br>Want / desire - have less priority;<br>Urge/craving - have higher priority;<br>Lacking confidence;<br>Awareness;<br>Choices	"When I have an urge to move, in my head I start hyper-focusing on it. I'm thinking, "Okay, what can I do after this moment". If I am stuck in class I'm thinking, "Okay, could I go work out? Could I go walk? Could I do this?" I'm thinking, "Okay, I am ready to go - now!" (9/21, A)<br><br>"A want for rest would be similar to you're in class and you want to sleep, but you have other things [to do]. It's the same thing. You just put it at the bottom of your priority list." (9/15, A)<br><br>Related quotes found above and below.
3	Behavioral regulation	Processes of managing behavior to achieve certain outcomes	11	Can't rest - need to be productive / do work / play sport;<br>Conscious decision making to weigh the motivation states;<br>Should / Need to recover and prioritize rest;<br>Change behavior patterns;	"If I meet everything, and I'm gonna do this, things will be fine, but then also because I am literally exhausted." (8/31, D)<br><br>"After making that decision of wanting to rest, [I thought], "Oh, if you rest, or if you move, nothing really stops", and so, those conscious, decisions of, "How am I feeling now? And what will come in the future? And what should I do? What would be more beneficial for me, to prepare myself for the future?" (9/10, B)<br><br>"Right now I am thinking about what homework I am doing when I am done with this interview - instead of thinking about getting ready for or going to bed tonight." (9/21, C)<br><br>"I just got into running, and I was thinking, "okay, tomorrow morning, two-mile run okay?" [And then] I didn't do it. I stayed in bed, I was thinking, "oh, I think I'm gonna sleep an extra hour". So that was my failed attempt to move. But yeah - I had a plan. I was thinking, "okay, 7am, wak[ing] up" [laughs], and "no I, I'll sleep until 8:00, I have time" [laughs]… and, I don't know, I think I was just really, tired and overwhelmed." (9/21, B)
4	Anticipation/ energy mgt.	Looking into the future for upcoming energetic demands and recovery	10	"Psyching" the self up / "pumping up";<br>Anticipating the next thing to do;<br>Reserving energy for the next task;<br>Knowing that planned physical activity is getting closer;<br>Knowing you have a full day ahead;<br>Looking forward to exercise;	"I definitely don't feel as much of an urge to rest, definitely more energized now that we're talking. I enjoy this [laughs], so, [it] makes me feel good and also it's 2:16pm. I have another tour to give so subconsciously I'm pumping myself up…just to get ready for the next thing that I have to do." (9/27, A)<br>"I will tell myself, 'You know you have this long until this assignment or whatever is due, and you **need to get up** and get that done or you know you'll get a percentage taken off, or the points taken off, and you don't want that' so it's kind of like, trying to give myself a pep talk to get myself

				Managing energy / rationing or conserving	up to move. And sometimes it works, but if it doesn't, I will rest just a little bit longer, and then I'm like "ok, **<u>you gotta get up</u>** and go." (9/13, A)<br><br>"I try to save my moving for that period where I have to be really active and engaged." (9/21, A)<br><br>"I've been anticipating practice [for sports]." (9/10, A)<br><br>"I think what makes me want to rest if what's next to come, just in preparation - anticipation. Mentally preparing or mentally resting for whatever's next to come in my schedule." (9/10, B)
5	Cues / triggers / feedback	Effect of stimuli on motivation states	8	Fitness tracker feedback;<br>Desires / wants - triggered by a cue or event;<br>Negative emotions -> want to move more (to get rid of stress);<br>Positive emotions -> want to move more (on a win streak);<br>Music (lyrics)	"[I will read] 'here are the top 10 exercises you should be doing to work on getting rid of your stomach fat' or 'trimming down your thighs', and I will read those articles and [will think] 'oh, you know, what are those exercises?' and then I will instantly [think] 'Oh, I should try these, I should be doing this'. (9/27, A)<br><br>"When things are going well you want to keep playing and keep on the field" (9/17, A)<br><br>"When I'm getting ready to go out [to socialize], and I finish, [I see myself in the mirror, and] I think, "Oh yeah, I look, I look okay!", and then it makes me wanna go out and do stuff." (9/8, A)
6	Willpower / self-control	Resistance provided by the self in the face of desire	4	Depending on willpower / forcing self to do something;<br>Getting psyched up;<br>Wandering / unfocused mind	"I think when I have wants for rest, I usually, even if I have other priorities like homework, I'll give in to it, almost too easily. I will find myself scrolling on my phone when I'm supposed to be doing homework. It's kinda hard not to give in." (9/21, D)<br><br>"I am trying to get [my desire] back up so I can actually work out" (9/21, A)<br><br>"Sometimes I desire to rest because I don't let myself get enough sleep." (9/13, A)
7	Consummation / Behavioral enactment	Actual engagement in the focus on one's desire	2	Took a nap;<br>Worked out this morning	See Table 5 above.
			TOTAL = 64		

* * These are the number of LOTs originally attributed to this HOT theme by analysts. During stages of re-review, some LOTs were reassigned to different HOTs for coherence, which may slightly change the quantity of LOTs in the following column.

** Many LOTs can (and may be) cross loaded onto other HOTs.

*** Many quotes can be cross loaded onto other HOTs, but efforts were made to place unique quotes only into 1 (or two) HOTs.
