# Supplementary material for: Qualitative and quantitative evidence of motivation states for physical activity, exercise and being sedentary from university student focus groups
Source: Front Sports Act Living. 2023 Mar 21;5:1033619. doi: 10.3389/fspor.2023.1033619 (PMC10071436; doi:10.3389/fspor.2023.1033619)
Supplement: Supplementary file 4 [file Table4.pdf]

Supplementary Table 4. Super Higher Order Theme 4: Objectives and impulses

| # | Higher order theme (H.O.T.) | Explanation | L.O.T.s attributed to this H.O.T. (count) * | Exemplar L. O. T.s ** | Exemplar Quotes *** |
|---|---|---|---|---|---|
| 1 | Goals | Desired endpoint, condition or achievement | 29 | "Have to" or "should" - based on a goal;<br>Goals - more like "force myself";<br>Move to achieve a goal;<br>Goal to be healthy results in motivation for exercise;<br>Goals to develop confidence, energy, get into a routine, be healthy, be productive, lose weight, improve appearance, etc.;<br>Movement goal;<br>Achieved goal;<br>Was productive;<br>Inspired to move;<br>Aspirations to move;<br>Had bad experience and wanted to improve;<br>Start planning about possibility for the future;<br>Self-improvement | "I see the exercise[s] as something I can achieve - and my goal at some point down the line. That is usually the main moment where I think, "I very much want to move." (9/27, A)<br>"[What makes me want to move is] more like step count, you know? It's like, 'I haven't reached my goal yet'… because you can't see it go down the next day, you know?" (8/31, D)<br>"I don't want to heavily exert myself because I have already done that today, and I have checked that off my list." (8/31, D)<br>"For me it would definitely also be, about, keeping, myself healthy…" (9/17, B)<br>"… I would say that that moment, yeah, that moment I just felt inspired to, to move, all aspects and change my, life around." (9/10, B) |
| 2 | Drive | Internal force to move or sleep, similar to hunger, thirst, etc. | 18 | Activity quota: Must get some activity in every day (internal need for it);<br>Drive to sleep;<br>Drive to recharge and recover;<br>Having hyperactivity / having a burst of energy;<br>Understimulation;<br>Need to move;<br>Urgent movement into action | "Each day I need to be active, but then I rest because of the activity." (8/31, D)<br>"I need to sleep - now." (8/31, D)<br>"… it's a natural thing [where] you crave sleep because you can't really just stay up all the time because you need to sleep. It's more primal, I guess. Because everyone has to sleep. You don't have to move. Well, I guess it depends. There are people who don't really move. But there are some people that do. But everyone sleeps, no matter how active you are." (9/15, A)<br>"I feel like I'm not doing anything - so I wanna get up and move." (8/31, B)<br><br>"I think that want and desire as more like a superficial thing. It's not going to last. It's short term, but then urge or crave is almost like you physically need to." (9/17, C) |
| 3 | Have to/ need to/ should / must | Extrinsic forms of motivation, such as external and introjected regulation | 12 | Commitments - have to run<br>Shoulds;<br>Shoulds / "have to's" can undermine wants / desires;<br>Motivation states similar to a "need to";<br>Motivation states similar to a "should";<br>Motivation states feeling like guilt for not moving; | "I feel like I shouldn't move because it would distract me from my emotions, basically. If I am really sad I use running as an excuse to continue to be in that sadness, instead of facing what's actually making me sad." (8/31, D)<br><br>"… [I am thinking], "okay, I've earned, the right to go to sleep, I've earned the right to rest", and now my body will actually let myself rest without me [thinking] "I still have stuff to do, I can't relax" [laughs] "I can't rest". (9/27, A)<br><br>"I guess a want to move is when you know you haven't exercised in a while and you know you probably should and you really want to, but it's not enough to actually do it. You put it on your priority list, but it's at the bottom." (9/15, A)<br><br>"I urge myself to move because I know that I need to - because I am either sitting down all day in classes, and I know **I need to** get myself to walk around and get some fresh air or just experience all of the things around me." (9/13, A)<br><br>"If I want to move, that is usually me laying on the couch feeling bad about myself [for not moving], but I also don't really have any desire to move. The want is 'eh, I should probably do this, because I know its good for me', but I don't actually do it" (8/31, D) |

| | | | | | |
|---|---|---|---|---|---|
| 4 | Instrumental demands | Movement and rest as having utility and something you must do in life, mostly mindlessly | 12 | Movement as instrumental - have to move to get to places;<br>Movement as instrumental only;<br>Can't move: Have to be sedentary due to task at hand;<br>Have to move: build environment forces you to move;<br>Have to move: athlete<br>Have to move: Performances/ rehearsals / work activity<br>Movement you are paid to do / Labor - not internally motivated to do;<br>Utility of movement | "You walk in order to get someplace. You don't walk to enjoy it, unless you're doing it to enjoy it." (9/27, A)<br><br>"There's movement I do out of utility." (9/17, A)<br><br>"I am taking notes so I can't move during that." (9/21, C)<br><br>“I'm not motivated to do [the walking], but I still do it because you have to do it for the [job]." (9/27, A)<br><br>“I’m on cross country, so I have practice every morning, so I absolutely like have to [move and be active].” (8/31, D) |
| 5 | Conflict | Desires impinging on each other | 10 | Want to move but will rest when tired;<br>Conflict / competition of different desires occurring at the same time;<br>Conflicts between desires to rest and move;<br>Torn between two decisions;<br>Want to move and urge to rest at the same time - busy day;<br>Motivation states strong enough to override other desires;<br>Considering future commitments; | "Some days, coming home from work **<u>I want to rest</u>**. There are some days **<u>I want to move as well</u>** because I work mornings sometimes on Sundays, and I come back and I'm like, "Oh, well I have stuff to do. I **<u>need to move</u>** and get that done", but I can't because I'm tired in some ways, but I **<u>know I need to get them done</u>**, so I can't. It's a **constant battle between the two of them**, and I don't know how to combat either one of them. I will do a little bit of both move and rest. I will sit and give myself a time limit to rest, and then **I will force myself to get up**. I will tell myself, "You know you have this long until this assignment or whatever is due, and <u>you **need to get up**</u> and get that done or you know you'll get a percentage taken off, or the points taken off, and you don't want that" so it’s kind of like, trying to give myself a pep talk to get myself up to move. And sometimes it works, but if it doesn’t, I will rest just a little bit longer, and then I'm like "ok, **<u>you gotta get up</u>** and go." (9/13, A) |
| 6 | Secondary desires | Desire to move or rest only as secondary to some other desire that is important. | 5 | Want to be [more] productive;<br>Need to get to event / class / appointment on time;<br>Move for sport;<br>Not being productive and doing nothing | “… if I’m [feeling] the urgency to [move], it’s like “ah!”. You know that, “prom is about two months away, I need to get started and get in shape now - gotta fit into that dress.” (9/27, A)<br><br>“… I wanted to begin, cleaning my room, reading my book for a class, [I was thinking] “I’m awake, I should be moving”, but, because I was, hung over and confined to my bed, where I needed my eyes to be closed and a pillow over my head, I couldn’t satisfy the urge to move and get stuff done.” (9/27, A) |
| 7 | Temptation | A desire that conflicts with something “you should do” or a value that you have | 1 | Sometimes, but not always, giving into cravings | Participant C: "I only crave rest right after I wake up, because I feel that as soon as I get going in the day its fine. If I actually get myself up, the craving for rest goes away. So, I will wake up and its, "Oh my god it's 7am. All I want is to go back to the bed. And then as soon as I go brush my teeth or something, I'm thinking, "What was I tired for?" and its fine. I woke up that way essentially, and the craving goes away, and I am fine for the rest of the day."<br>Interviewer: “Have you, do you ever satisfy that craving, when your alarm goes off at 7:00 [and “I’m so tired”]…”<br>Participant C: Absolutely [laughs].<br>Interviewer: “…and then you go just go, like “I’m not doing this” and you go back to bed?”<br>Participant C: “Sometimes, yeah…”<br>Interviewer: “Okay.”<br>Participant C: “…but if I like actually get myself up it goes away.” (9/21, C) |
| | | | TOTAL = 87 | | |

* These are the number of LOTs originally attributed to this HOT theme by analysts. During stages of re-review, some LOTs were reassigned to different HOTs for coherence, which may slightly change the quantity of LOTs in the following column.

** Many LOTs can (and may be) cross loaded onto other HOTs.

*** Many quotes can be cross loaded onto other HOTs, but efforts were made to place unique quotes only into 1 (or two) HOTs.
