# Supplementary material for: Qualitative and quantitative evidence of motivation states for physical activity, exercise and being sedentary from university student focus groups
Source: Front Sports Act Living. 2023 Mar 21;5:1033619. doi: 10.3389/fspor.2023.1033619 (PMC10071436; doi:10.3389/fspor.2023.1033619)
Supplement: Supplementary file 5 [file Table5.pdf]

Supplementary Table 5. Super Higher Order Theme 5: Propelling and restraining forces

#	Higher order theme (H.O.T.)	Explanation	L.O.T.s attributed to this H.O.T. (count) *	Exemplar L. O. T.s **	Exemplar Quotes ***
1	Facilitators of movement	Internal and within the person's control<br><br>AND<br><br>External and within the person's control	40	Caffeine;<br>Day off - want to go to gym;<br>Environment / being outside;<br>Music - beat and/or lyrics;<br>Music - puts you into a good mood;<br>Social media / gym selfies / motivational videos;<br>Video game - excitement / adrenaline rush;<br>Wearable fitness technology	"For me music is what impacts my urge to move or rest, because if it's slow, sad music I just wanna chill, and go for a drive or something, but if it's up-beat I will dance or [laughs]…or I will go work out…" (9/21,D)<br>"I have a lot of friends on social media who will post gym selfies, and when I see those I'm feeling like, "Ah, look at them. I should probably do that!" That's an outside factor that potentially pushes me to want to move or do what they are doing. It never really happens, but definitely I mentally get that, but not physically." (9/27, A)<br>"I used to play basketball and soccer in high school so when we had a dope mixtape before a game, I always felt, "Let's get hyped. Let's go into this." Whereas, sometimes my coach would play country music, and no one wanted to do the [sport drills], because it was country music, [and] how are we supposed to hype up? If you don't like the music, how are you gonna do anything? I had a teacher who used to play country music, and we'd say, "You gotta change the music, we're not doing anything." (8/31, B)
2	Deprivation / satiation	Scarcity of movement or sedentary behaviors compared to excess and "fullness"	32	Been sitting a long time;<br>Too much sitting;<br>Wanted to move around after a long, 7-hour road trip;<br>Was sitting two hours;<br>Move after sitting in class;<br>"Cooped up" during winter;<br>No exercise / training for an entire week (e.g. Spring break);<br>No exercise the previous day;<br>Lack of sleep and need to recover;<br>Physiological deprivation of sleep (don't get the 8 hours needed);<br>Sleep deprivation;<br>Already rested;<br>Fully rested;<br>Just exercised;<br>Already moved a lot	"...there are other days where it's a craving to the point where, you're dying to get a hard workout in - you really miss it and wanting to train and push yourself." (9/17, A)<br><br>"[On] those days that I go to bed at midnight and then wake up at 6am I just want to go right to sleep right after class." (9/10, A)<br>"I think I have the urge when something is going on in my life where I just need to get out, and I need to run if I have been sitting for a long time. I need to just run on vacations. We would always stop at a rest area for a road trip, and I would literally just get out because when I was little I'd just run to the playground. I needed to run because I craved moving because I was in the car for about 10 hours." (8/31, D)<br>"… after a long time of movement you want to rest, but [after] a long time of resting, you don't want to get up and you know, run two miles." (9/8, A)<br><br>"Over the past week I have wanted to rest more than usual. We had a pipe burst in the dormitory so I was up for about 5 hours from 11pm to 4am. I was dying because I was on my feet for that extreme period of time. I wanted to recover but haven't been able to so that experience really made me want to just lay down and not do anything." (9/21, A)
3	Barriers for movement	Conditions and situations internal to the person, NOT in their control	31	Illness;<br>Pain causing dread of movement;<br>Surgery;<br>Injury – can't move;<br>Poor diet;<br>Feel stuck / trapped	"Craving rest - I would probably say for me the only time I really feel like that is when I'm sick or something like that. When I had COVID-19 at the beginning of last year I just felt run down. I was thinking, "All I want to do is just rest". I had ample time to do so. I was stuck in my place by myself [quarantining], but that was a craving where it felt like an itch that needed to be scratched. It couldn't be pushed off." (9/17, A)<br>"I have problems with my knees, so I'm kinda scared of starting." (9/8, A)<br><br>"Last summer, I [had] cataract surgery and the recovery period was longer than I expected, and since my procedure was [more complicated] I had to sit in bed and get help to walk to the bathroom for a week. And I couldn't drive my car for three weeks, and I couldn't lift anything over twenty pounds for two months, and I just wanted to move so badly, but especially that first week, because I literally couldn't even go up and go walk around the house or anything…" (9/17, B)

4	Social influences	Effects of people, lack of people, and social situations	30	Social influences;<br>Social reasons;<br>Social pressure;<br>Watching others move or be sedentary;<br>Friends / Peers / Significant others;<br>Coaches and peers pushing you;<br>Too much interaction with others – want to rest;<br>Social media;<br>Effects of isolation;<br>Being alone;<br>Seclude self from people;<br>Too much socializing;<br>Long day at work and want to be alone;<br>Vicarious experience	"I have very high social needs, and so I feel that if the people I'm around are all going to be active, then I feel a need to do that too because … that's not how [I] fit in, but that's how I can be with them, and get my social needs and interaction for the day." (8/31, D)<br>"There's definitely moments of time where I am in my dorm room thinking, "oh man". I'm just studying, doing my work, but I feel that I can be with my friends. We're having a great time, but then my social battery runs out and I'm thinking, "oh, I should probably get some rest" for the next day." (8/31, B)<br>"We'll finish a game, [maybe] the next day we have off, I'm like "oh, I wanna go to the gym". He's like, "I'm wiped out from yesterday, I don't wanna go to the gym", and sometimes, him not wanting to come with me, that makes me like "eh, well do I really need to go?", it makes me, often times I still will go anyways but it definitely gives me more of a hesitation than if he's like, "yeah let's do it" then, it's "boom, let's go right away". (9/17, A)<br>"When I get overwhelmed, I prefer to rest and just being alone resting." (9/10, C)
5	Conditions	Conditions and situations external to the person, NOT in their control	29	Weather;<br>Good weather is refreshing and promotes good mood;<br>Bad weather - afraid you might slip or not be safe;<br>Bad weather puts you in a bad mood;<br>Hot weather - instigates wants to stay inside with comfort (A.C.);<br>When forced to be outside in bad weather, go faster to get inside sooner;<br>Conditions must be right;<br>COVID-19 quarantine;<br>Too crowded	"The weather is something that definitely urges me to move. Morning sunrises or evening sunsets - those are definitely something that urges me to move, go on runs or walks because, obviously for the scenery, but the breeze as well. It's a pretty scene to see." (9/13, A).<br>"I feel that want and desire is when I want [to move] but can't because I have responsibilities at home. That's mostly when I watch my nephew after school or on weekends. (8/31, B)<br>"If I do the exercise it depends on where I am at. If I am in my room and there is no one around and I have time, absolutely, I will bring out my yoga mat, and I will [exercise], but if I am in the dining hall, I can't instantly act on my desire to move." (9/27, A)<br><br>" … back to cheerleading, we weren't allowed to stop, because of COVID-19, [so] we weren't able to, do that, and that's something that I did a lot during high school was [stunting], so I always had an urge to want to, [stunt] and put people up in the air but I wasn't able to…" (8/31, C)
6	Liking / reinforcement	Rewards and pleasure from movement and sedentarism	17	Enjoy exercising;<br>Hedonic pleasure/ liking;<br>Desires are related to enjoyment;<br>Like to move (feel refreshed);<br>Like the post workout "after glow";<br>Movement has psychological rewards;<br>Moving felt really good so want to keep moving;<br>Wanting the adrenaline high from movement;<br>Joy of movement;<br>Reward after accomplishment	"What makes me want to move is just the joy I get from playing sports. I enjoy exercising [and] definitely feel motivated…" (9/17, A)<br>"Soccer playing and training is the most enjoyable part of my day." (9/17, A)<br>"Moving and walking outside is mentally refreshing." (8/31, D)<br>"I really enjoy starting my day off because then I feel like really refreshed, so I guess, in the mornings I really wanna be active." (8/31, D)
7	Immersion	Flow states of high focus	5	Highly focused on the desire during craving;<br>Engaged with interview (i.e., flow state);<br>Engaged;<br>Have a strong interest in a sedentary activity (like interesting schoolwork)	"I am pretty engaged with this interview right now, so I don't feel the need to move." (8/31, A)<br>"If I am studying I don't feel the need to move because I am doing something that interests me." (8/31, B)<br>"If I am distracted, such as studying, and I am completely immersed in it, then I won't feel the need to move around. I will rest for a little bit and do what I need to do." (9/10, A)
8	Distraction	Lack of focus	1	Paying attention to other things	"… if I'm aware or bored, then I just wanna start moving around. So if I'm in a lecture, and I'm completely bored, I wanna start getting up and moving. I'm distracted." (8/31, A)
			TOTAL = 185		

* These are the number of LOTs originally attributed to this HOT theme by analysts. During stages of re-review, some LOTs were reassigned to different HOTs for coherence, which may slightly change the quantity of LOTs in the following column.

** Many LOTs can (and may be) cross loaded onto other HOTs.

*** Many quotes can be cross loaded onto other HOTs, but efforts were made to place unique quotes only into 1 (or two) HOTs.
