# Supplementary material for: Qualitative and quantitative evidence of motivation states for physical activity, exercise and being sedentary from university student focus groups
Source: Front Sports Act Living. 2023 Mar 21;5:1033619. doi: 10.3389/fspor.2023.1033619 (PMC10071436; doi:10.3389/fspor.2023.1033619)
Supplement: Supplementary file 6 [file Table6.pdf]

Supplementary Table 6. Super Higher Order Theme 6: Stress and boredom

| # | Higher order theme (H.O.T.) | Explanation | L.O.T.s attributed to this H.O.T. (count) * | Exemplar L. O. T.s ** | Exemplar Quotes *** |
|---|---|---|---|---|---|
| 1 | Demands | External factors impinging on the self | 52 | Overworked<br>Being overloaded;<br>Heavy demands - very busy;<br>Heavy load, overworked;<br>Busy schedule;<br>Studying;<br>Too busy to move / exercise;<br>Too much traveling;<br>Increased life demands;<br>Demands from training / sport;<br>External regulation - sports training;<br>Increased training load / workload;<br>Situational demands: "have to" study, "have to" go to bed;<br>Want to rest, but can't : **have to** keep working in the lab; go to practice;<br>Period of focusing really hard: academic class or homework (mental challenges);<br>Move - job responsibilities;<br>Lack of breaks / recovery;<br>Want to keep exercising, but not physically able;<br>Decreased demands;<br>Coming home from work and now want to rest;<br>Just want to lay in my bed - had a long week | "I have had a jam-packed day full of stuff, and I am going to be going until 11pm - so it's a bit more like jolty, anxiety movement. I want to kick my foot around a little, or shake a bit, just to get rid of that nervous energy, in terms of moving, in terms of exercise or working out." (9/27, A)<br><br>"When I used to cheer there was a week we have practice every single day, and then we ended with two competitions, and they didn't end well. They were really bad, so at the end of the week you're just craving to rest and sleep, and mentally drained too, versus, "Oh, I had practice this week, and I'm tired at the end of the weekend - I want to rest." (8/31, C)<br><br>"One of my classes got cancelled and moved online, and then I didn't really have much to do - so today was more of a lazy day for me. I took a nap." (9/21. A)<br><br>"I really wanted to go get a good workout, and I was all prepared. I was, "okay, I'm gonna go work out for an hour, get a good run in, lift some weights" and just feel really good about myself, and then I realized how much work I had to do for school, and I just can't leave. There's not enough time in the day to do both, so I had to make the decision to stay in my dorm and work on stuff - stuck at my desk ..." (9/21, A)<br><br>"I just wanted to rest, but of course I was unable to." (9/15, A) |
| 2 | Physical sensations | Psychosomatic sensations, like energy and fatigue | 37 | Physical sensations;<br>Urges feel physical in nature;<br>Urge is physical / of the body;<br>Differences between physical and mental sensations;<br>Tired;<br>Just woke up;<br>Exhausted / exhaustion;<br>Feelings of energy;<br>Bursts of energy;<br>Want to expel excess energy;<br>Adrenaline rush after moving;<br>Feel a tension that you want relieve from, and will move to get rid of tension;<br>Feeling antsy now;<br>Feeling jittery;<br>Feeling groggy;<br>Over-eating (feel gross, which is stressful);<br>Nauseous / dizzy (due to hangover);<br>Observable manifestations (drooping body, stop talking);<br>Urge - fidgeting, twitching starts;<br>Nervous / jittery | "... The urge to rest is more of a physical thing [like feeling] really tired from walking all day. My body feels forced to rest." (9/17, B)<br><br>"The desire to rest is more motivated by my body and how my body is feeling and the desire to move is more like a mental thing." (9/17, A)<br><br>"I just walked here, and I got a 20 minute walk in - so I am just very energetic right now." (9/10, A)<br><br>"I get like random bursts of like energy, and it makes me really motivated, and it like makes me want to get more done, and it makes me wanna go on runs, go on walks, get more homework done, or get the next week's homework done." (9/13, A) |

| | | | | | |
|---|---|---|---|---|---|
| 3 | Stress | Deviations from homeostasis | 32 | Stress;<br>Stressful situations (unexpected, uncontrollable);<br>Stress (of the interview);<br>Stress (preparing for exam, speech);<br>Stress could make you less or more active;<br>Stress activates too;<br>No motivation to either move or rest - due to stress - possibly conflict;<br>Stress stimulates motivation states like craving;<br>Excitement of competition and winning;<br>Strong desire to move at football game - exciting atmosphere - winning the competition;<br>Overstimulation - a lot of excitement;<br>"Stressed out" and overwhelmed;<br>"Stressed out" / overwhelmed / emotional exhaustion due to concerns about past and future;<br>Urge to move and/or rest when stressed out;<br>Coping / dealing with the want to rest;<br>Stress and disappointment;<br>Feel highly agitated and frustrated; aggravating, annoyed or stressed; because can't satisfy the urge | "Stress makes me want to move, but if it's too much there's a certain point where … if I don't have any breaks to sit for a minute … then I feel like I need to go home and rest at the end of the day." (9/17, B)<br><br>"When I get stressed I am "Go! Go! Go!" I get these bursts of energy. Sunday night I was super stressed, and so at 10pm I just cleaned my entire dorm room, my bathroom, my bed, my closet, refolded everything. I am the exact opposite of most people who will get down and be like, "oh, I'm just gonna go lay down, chill out." I am, 'Let's do 40 tasks right now". (9/21, C)<br><br>"When I am feeling stress or anything like that, always, working out is what I turn to. I will skip something else in my day to get a workout in. If I am really feeling overwhelmed or stressed or whatever it is … because I know how much [exercise] alleviates that [stress] for me" (9/17, A).<br><br>"I think my [desire to rest] is related to stress. If I get really stressed out, my brain will shut off, and I just need to sit and not do anything. Otherwise, if I am thinking too hard I am not doing the work correctly because I am overthinking. So [my desire to rest] is normally about stress and if I am freaking out I just need to sit down." (9/21, C)<br><br>"I tend to shut down when I get stressed or overwhelmed." (8/31, C)<br><br>"I couldn't satisfy the urge to move and get stuff done, and that was very stressful. It was annoying, and I was saying to myself, "Why won't this go away!" (9/27, A)<br><br>"There's just no motivation, and I am just stressed." (8/31, C)<br><br>"…during basketball we'd be in the gym for hours every day, going home at 8pm and then the games - there was always the game that you lose. Sometimes it is not that big of a game, but sometime it could be a state championship, and you just crave rest, mentally and physically. You've put in so much effort, just to lose." (8/31, A)<br><br>"There are times when I don't wanna face it, but then when I do face it, it all hits at once, and I get completely overwhelmed by it. I just need to like take a seat, and just reset myself, maybe go on my phone for a good little bit, go on a walk, and, I used to walk out of my house, if I had to… go on a walk around the neighborhood." (8/31, A) |
| 4 | Exhaustion threshold | Point at which fatigue has a substantial effect on motivation states and behavior | 32 | State of exhaustion;<br>Mental exhaustion;<br>Physical exhaustion;<br>Being "run down";<br>Brink of exhaustion;<br>Exhaustion resulting in cravings;<br>Overpowering craving for rest and sleep due to heavy socializing;<br>Exhausted from constant traveling and just want to rest;<br>Exhausted / overwhelmed;<br>Stimulus overload;<br>Fatigued | "..When I reach the brink of exhaustion, that's when I personally wanna rest as a college student, because my day's just so jam-packed with meetings and classes … What makes me wanna rest is when my body is just so exhausted I can't continue anymore…" (9/27, A)<br><br>"I am just too tired. I have wanted to be active, but I just don't always have that energy." (9/10, A)<br><br>"When I am exhausted, that is when I literally can't anymore. I need to just lay down. At the end of the season where I have been training, forever, and mentally racing - it's very mentally taxing. So it's when I'm exhausted and physically and mentally exhausted where I just crave a break and rest. I think that's when I [have] the highest amount of urge." (8/31, D) |

| | | | | | |
|---|---|---|---|---|---|
| 5 | Monotony / Boredom | Sensations of being underwhelmed, under stimulated | 12 | Need for change;<br>Need for stimulation;<br>Boredom;<br>Monotony / high repetition;<br>Boredom results in / triggers desires to move;<br>Bored;<br>Bored with current activity;<br>Would be more bored with rest / sedentarism | “What makes people want to move is a change after doing the same thing over and over which can be monotonous for a person and a little boring." (9/10, B)<br><br>"I think that on a global scale, what makes people want to move is a change after doing the same thing over and over which can be monotonous for a person and a little boring. Maybe that change of movement could [result in] the after-effects of just moving the body" (9/10, B)<br><br>"For me, something that really does make me want to rest is when I'm doing something repetitive." (8/31, B) |
| 6 | Emotion regulation | Ability or capacity to modulate feelings | 11 | Alleviate stress;<br>Reducing stress;<br>Move to combat stress;<br>Clear mind / emotional stability;<br>Clear mind and thinking;<br>Feeling aggravated;<br>Feeling frustrated;<br>Bad mood results in cravings;<br>Move for a better mood;<br>Feel a need to relax and focus | "I notice on days that I am not active I am emotionally a lot less stable. [Activity] helps stabilize that. If I am absent [mentally] and all over the place [mentally] I think, "did I do anything today?" And then I exercise and it resets me and I can face anything." (8/31, D)<br><br>“… When I move around, it kinda clears my mind because I think a lot. So going to the gym or going on a walk just really helps me mentally…So I think that’s what makes me wanna move…” (9/21, B) |
| 7 | Mood / emotion | Positive or negative feelings and mood conditions, varying over a period of several hours | 3 | Good mood promotes desires / wants;<br>Move when in a positive mood state;<br>Feeling upset about trauma results in motivation states to exercise;<br>Feeling depressed and “bad frame of mind” results in wanting to lay in bed;<br>Feeling negative mood about body image problems leads to desires to exercise;<br>Feeling agitated and/or annoyed | "I want to move normally when I have those days where I wake up in a really good mood. I want to move and get everything done and walk around more, run, exercise, etc." (9/13, A)<br><br>"I experience desire when I struggle with my body image. Rather than doing something drastic in a negative way, like changing how I am eating, I would rather exercise because it is a healthier option. I do a healthy amount of exercise when I feel bad about myself and I don't over-exert myself." (9/17, B) |
| 8 | Dysfunction | Deviations from homeostasis that result in conflicting signals and impaired ability to act as intended | 1 | Hungover;<br>Mania related to bipolar disorder / hyperactivity | “I was a bit hungover, and I was stuck to my bed because I was a bit nauseous but, I [couldn’t] fall asleep. [I thought] “If you can’t rest, you should be doing something”, and, it was very, annoying because I wanted to begin, cleaning my room, “I’m awake, I should be moving”, but I needed my eyes to be closed and a pillow over my head, I couldn’t satisfy the urge to move and get stuff done, and that was very stressful.” (9/27, A)<br><br>"It would be similar to an urge I guess too. I have bipolar disorder so it makes me manic at times, so that fuels that [desire/want] if I am manic. I guess that makes me different from most normal people as they want to move, or have an urge to move, but I kind of have to move." (8/31, A) |
| | | | TOTAL = 180 | | |

* These are the number of LOTs originally attributed to this HOT theme by analysts. During stages of re-review, some LOTs were reassigned to different HOTs for coherence, which may slightly change the quantity of LOTs in the following column.

** Many LOTs can (and may be) cross loaded onto other HOTs.

*** Many quotes can be cross loaded onto other HOTs, but efforts were made to place unique quotes only into 1 (or two) HOTs.
