# Supplementary material for: Qualitative and quantitative evidence of motivation states for physical activity, exercise and being sedentary from university student focus groups
Source: Front Sports Act Living. 2023 Mar 21;5:1033619. doi: 10.3389/fspor.2023.1033619 (PMC10071436; doi:10.3389/fspor.2023.1033619)
Supplement: Supplementary file 7 [file Datasheet1.pdf]

Supplement 1. Focus group interview questions

Q1 - How much have you wanted to *move* over the past week?
Q2 - How much have you wanted to *rest* over the past week?
Q3 - Do you want to *move* right now?
Q4 - Do you want to *rest* right now?
Q5 - What makes you want to *move*?
Q6 - What makes you want to *rest*?
Q7 - How do you experience motivational states related to *movement*?
Q8 - How do you experience motivational states related to *rest*?
Q9 - Describe a recent time you had a desire, want, urge to *move or rest*.
Q10 - How do external factors impact urges to *move or rest*?
Q11 - Describe a time when you had a strong urge to *move or rest*, but were unable to satisfy that urge.
Q12 - Do you feel an enhanced urge to *move or rest*, compared to the start of this interview?
